# Quasi-experimental study on the effectiveness and impact of implementing nurse-led ‘therapeutic optimisation’ (THEO) intervention in two older persons wards: a mixed methods study protocol

**DOI:** 10.1136/bmjopen-2025-102529

**Published:** 2025-08-26

**Authors:** Yetunde Ataiyero, Alisen Dube, Joanne Odell, Vanda Carter, Hazel A Smith, Sally Hardy, Alison Leary, Sarahjane Jones

**Affiliations:** 1 Centre for Health Innovation, University of Staffordshire, Stafford, UK; 2 University of East Anglia, Norwich, UK; 3 Centre for NMAHP Research and Education Excellence (CeNREE), University Hospitals of North Midlands NHS Trust, Stoke-on-Trent, UK; 4 London South Bank University, London, UK

**Keywords:** Nursing Care, Patient Reported Outcome Measures, Patient Satisfaction, Patient-Centered Care, Nursing research, Work Satisfaction

## Abstract

**Abstract:**

**Introduction:**

Higher staffing levels, particularly with experienced registered nurses, are associated with improved patient safety and fewer adverse events, as skilled nurses can quickly identify potential risks and implement strategies to mitigate them, creating a safer environment for patients. This study will investigate the effectiveness and impact of implementing therapeutic optimisation (THEO) intervention, a complex intervention aimed at enhancing nursing care by increasing experienced registered nurse staffing and facilitating practice development (PD) activities for all staff.

**Methods and analysis:**

A multicentre quasi-experimental (before and after) study with an embedded convergent mixed methods process evaluation in older persons’ wards across two National Health Service (NHS) Trusts in England. Four work packages are proposed. Work Package 1 will use participatory action research to implement the THEO intervention, including an enhanced staffing model and PD activities, involving seven iterative participatory data collection exercises with staff, patients and their personal consultees, as appropriate. Work Package 2 will extract and aggregate anonymised administrative data (patient and staff related) from 1 January 2015 to 30 days after the 12-month intervention period. Work Package 3 will use qualitative interviews to explore the experiences of patients with and without mental capacity (with their personal consultees) and staff regarding the THEO intervention. Work Package 4 is a mixed methods process evaluation to assess implementation and contextual factors impacting the effectiveness of the THEO intervention, collecting both quantitative (survey) and qualitative (guided discussions and workshops) data.

**Ethics and dissemination:**

The UK Health Research Authority provided governance and ethical approval (Integrated Research Application System, IRAS ID: 334473). Dissemination will be with key stakeholders (including patient and public representatives), conference presentations and publications in peer-reviewed high-impact journals.

**Strengths and limitations of this study**

- This study combines a quasi-experimental design with an embedded convergent mixed methods process evaluation to explore both outcomes and implementation processes.
- It employs participatory action research to support inclusive, practice-based implementation and coproduction of knowledge with healthcare professionals.
- The study incorporates both staff and patient perspectives, including those with limited mental capacity, using ethical and context-sensitive methods.
- The embedded nature of the intervention across two different National Health Service Trusts allows for real-time learning but may introduce variability in implementation fidelity.
- Findings may have limited transferability beyond older persons’ wards, as the study is situated in a specific care context and population.

## Introduction

 The WHO 2016 Global Strategy on Human Resources for Health highlights the critical role of the healthcare workforce in building community and health systems resilience, thereby enabling effective response to disasters.^1^ Investment in the workforce is imperative for improving health, reducing vulnerabilities and ensuring preparedness for emergencies and climate change adaptations. The vision for 2030 is universal access to health workers for all communities, free from stigma or discrimination, requiring effective policies and significant investment. WHO projects the creation of 40 million new health and social care jobs globally and the need for 18 million additional health workers to meet future demands.^1^

Workforce shortages are severe, with potential for healthcare worker shortages affecting nursing professions, healthcare staff and general medical practitioners, with workforce gaps, now being described as workforce ‘gulfs’.^2^ Despite a significant increase in the UK NHS staffing over the past decade, the growing healthcare needs of the population continue to outpace workforce growth. Demographic pressures, an ageing population and changing disease burdens are driving increased demand for healthcare services. The COVID-19 pandemic exposed significant workforce challenges in the UK Health and Social Care service models, emphasising the need for workforce resilience and organisational support to prevent high resignation rates.^3^

At the start of 2023, older adults (65 years and older) were the primary users of inpatient hospital beds, had prolonged stays and were the most frequent users of health and social care services.^4^ An excess bed day, defined as each day a patient stays in the hospital beyond their expected length of stay for clinical reasons,^5^ costs the NHS between £2089 and £2532 per day.^6^ There is a wealth of evidence suggesting that the length of hospital admission influences the patient’s experience and ultimately their outcomes. The longer patients stay, the more likely they are to become deconditioned and have an increased risk of falls and hospital-acquired infections.^7^

Evidence suggests that higher staffing levels of registered nurses in hospitals are associated with better patient outcomes, including shorter hospital stays and reduced in-patient mortality.^8 9^ This is due to the higher level of training and expertise possessed by a nursing team with a greater skill mix and experience, enabling them to provide more effective and efficient care. Increased nurse staffing reduces patient adverse events, such as hospital-acquired infections and pressure ulcers,^10^ and lowers the incidence of falls, slips and trips.^11^ Moreover, higher nurse staffing levels improve clinical supervision,^12^ reduce medication errors,^13^ decrease readmission rates for patients with cognitive impairment^14^ and prevent staff burnout.^15^

Therefore, safe staffing levels involve assessing individual patient needs, evaluating ward factors, such as staff turnover, and considering nursing activities like clinical supervision.^16^ The National Institute for Health and Care Excellence recommends adequate nurse staffing to meet patients’ needs and identifies ‘red flags’ for staffing shortfalls. Given the clear link among hospital stay duration, patient outcomes and registered nurse staffing levels, this study proposes the therapeutic optimisation (THEO) intervention, a complex intervention package that aims to optimise nursing care in two NHS Trust wards by increasing experienced registered nurse staffing and facilitating practice development (PD) activities for all staff.^17^ PD is a complex intervention methodology that employs an emancipatory approach to guide person-centred, evidence-based healthcare. It involves engaging practitioners as active participants in change, using action-orientated and evaluation cycles to continuously inform and critique evidence, thereby sustaining new initiatives for safe and effective practices.^17^ Manley *et al*^17^ argue that the emphasis for PD remains on person-centred care, workplace cultures and systems, as well as working with complexity and conducting research ‘with people’ rather than ‘on people’.

### Study aim

To establish the effectiveness and impact of implementing the THEO intervention in older persons wards across two NHS Trusts in England.

## Methods and analysis

### Study design

A multicentre quasi-experimental (before and after) study with an embedded convergent mixed methods process evaluation to determine the effectiveness and impact of implementing a THEO intervention in two NHS Trusts.

### Study setting

Two NHS Trusts within the Norfolk and Waveney Integrated Care System in England. Norfolk and Waveney are considered high areas of deprivation and need.^18^ As a result, research has highlighted the need to have greater investigations on health inequalities that are present in coastal and rural areas.^19^ The clinical care setting will focus on care for older persons, which reflects the demographic population of Norfolk and Waveney. One care site is situated in a community NHS Trust, in a central, urban area of Norfolk, and the second site is situated in an acute NHS Trust in the East of Norfolk, identified as a coastal region.

### Study duration

The intervention will be delivered over a 12-month period in each site. While the overall study duration spans from February 2025 to May 2026, the two participating NHS Trusts will commence and complete the intervention at 3-month intervals due to recruitment schedules, within this window.

### Patient and public involvement

There was no patient and public involvement in the design of the protocol. However, patient and public involvement is embedded in the delivery of the study. The project management team and the project steering committee will include independent stakeholders to ensure that the study remains focused on patient and staff experiences and outcomes.

### Study intervention: Therapeutic Optimisation (THEO) of care

The THEO intervention aims to enhance care delivery through an enhanced staffing model and PD activities. This approach focuses on facilitating person-centred care, promoting safe and effective care and improving care coordination.^20^

Aligning with the NHS England’s Reducing Length of Stay Programme, the enhanced staffing model will provide additional experienced registered nurses to participating wards. This model emphasises clinical leadership, communication, support for best practice and embedded research and evaluation.^21^,^23^

Moreover, PD will be used to promote organisational change and person-centred care, thereby improving the quality of patient care.^24^ By engaging practitioners as active participants in any improvement, PD aims to optimise care processes and enhance clinical outcomes.^17 25^

Through the implementation of the THEO intervention, it is anticipated that evidence gathered will optimise effective care integration across the organisation and provide new knowledge on the impact of integration agendas and associated reforms across care levels in the Norfolk and Waveney Integrated Care Systems, ultimately providing people with universal quality care.

### Underpinning theoretical framework

Implementing health interventions is complex, involving the mobilisation of human, material and organisational resources to change practices within established settings.^26^ This study will use the realist evaluation (RE) framework, which links context, mechanisms and outcomes (CMO) discovered during project implementation. This theory-driven approach adapts to social realities,^27^ allowing for the exploration of complex research contexts. It identifies mechanisms that promote or challenge activities and captures outcomes for stakeholders.^28^ RE seeks to answer, ‘what works, for whom, in what circumstances and why?’ by examining generative mechanisms, contexts and outcome patterns.^27^

Using the RE framework allows for real-time insights into how changes occur, enhancing understanding and contributing to knowledge on innovation uptake in health and care.^29^ This framework will underpin the process evaluation of the THEO intervention to understand how the different components of the THEO intervention interact with each other, thereby influencing patient- and staff-related outcomes in study settings.

### Work package 1: implementation of the THEO intervention

#### Design

This work package will use the participatory action research (PAR) methodology to implement the THEO intervention. Participating wards will receive two additional experienced registered nurses who will provide direct patient care, actively participate in gathering and analysing evidence and work to improve the quality of care delivered on the participating wards. Seven iterative participatory data collection exercises will be conducted. These include a values clarification exercise (VCE), leadership assessment, workplace culture assessment, emotional touchpoint interviews, observations of care, fourth-generation evaluation and final participatory evaluation. Patients and their personal consultees will only participate in the emotional touchpoint interviews. The remaining exercises are for staff and co-researchers. The THEO PAR Handbook (see online supplemental material 1) outlines the principles of participatory approaches, ethical considerations, details of each activity and specifies who will be involved.

#### Objectives

The specific objectives of this work package are as follows.

1. Work with participants in collaborative, inclusive and participatory ways^30^ to implement PD activities with an enhanced staffing model.
2. Co-create knowledge to enable participants to understand patterns, habits and rituals contributing to workplace culture^22^ in the context of their practice.
3. Critically reflect and analyse the evidence to cocreate new knowledge.
4. Create shared action plans to enable informed actions.
5. Repeat the above objectives in action-orientated cycles throughout the implementation study period.

#### Study participants

THEO study participants will include patients (both with and without mental capacity) and staff members. There will be two broad staff groups.

1. The co-researchers comprising the THEO PD facilitator, THEO intervention registered nurses whose roles exist explicitly for the purposes of delivering the THEO intervention and self-selected or consensus-nominated clinical representatives from the unit’s multidisciplinary (MDT) team.
2. Staff participants are members of the workforce who work on the ward where the THEO intervention is being delivered but are not acting as co-researchers. Co-researchers will invite wider participation from the MDT team for specific data collection exercises. Staff participants may also include other Trust-based individuals who are key stakeholders but do not necessarily work on the ward.

#### Participant recruitment and consent

At the start of the THEO intervention, a generic study poster will be displayed on the ward to inform visitors and staff about the ongoing intervention. These posters will include contact details for the research team for those interested in learning more.

##### Co-researchers

The THEO PD facilitator and ward-based THEO intervention registered nurses will assume the co-researcher role based on their job descriptions. MDT representatives may self-select or be consensus nominated as co-researchers. Consent is assumed for the THEO PD facilitator, acting as the formal researcher. Although THEO intervention registered nurses are required to lead and facilitate THEO delivery in the clinical space, consent will still be sought for each data collection episode. Consent will also be obtained from additional co-researchers from the wider MDT. Research activities will be facilitated as a part of their roles in delivering service and promoting quality improvement.

##### Members of staff

Staff members providing direct patient care on the participating wards will interact with the THEO intervention. They will be notified when the intervention is about to commence, and when their wards become intervention wards through natural communication channels, such as team meetings and handovers by the ward manager. This group includes registered and non-registered nursing staff, allied health professionals and pharmacy staff assigned to and working on the intervention wards. Additionally, study posters will be strategically displayed on the ward informing staff members that it is a THEO intervention ward.

A single participant information and consent booklet will be shared with all staff members (including co-researchers), inviting them to participate in the study. The consent form in the booklet allows participants to sign up for as many data collection exercises as they wish by selecting each one individually. Because participants are being asked to commit to different exercises at the beginning of the intervention period, a withdrawal slip is provided for any aspects they might reconsider later. These are available as separate documents. A separate consent form allows participants to sign up for more aspects later if they wish and includes a ‘reconsent’ statement to indicate that they have previously consented to activities. Reconsent will also occur after any withdrawal to confirm consent for the remaining activities. The separate withdrawal form will permit participants to withdraw from more activities later.^31^

All participants will be asked before their participation in any data collection exercise if they remain happy to participate.

##### Patients and their consultees

###### Observations

Prior to commencing the observations, specific posters with key study information will be displayed around the ward to inform patients that a researcher may be present during their admission. These posters will provide at least 1-week notice of the observation date and time, summarise the study information and include contact details for the research team, should potential participants have any queries. Staff will inform patients, and where appropriate their consultees, when the researcher(s) will be present on the ward. Patients, and where appropriate their consultees, will have access to participant information sheets, which will contain all the important information to make an informed decision about whether to have their care observed, including QR codes to access an online participant information sheet.

Patients will be given the opportunity to verbally opt out of the study if they do not wish to have their care observed. For patients with limited or no mental capacity, their nominated professional consultees will be informed and provided with the study information sheet to make an informed decision about participation. Personal consultees will be prioritised over professional consultees when possible. If not, nominated consultees, such as a doctor or a staff member from another ward, will be sought to ensure independence from the research study.

All verbal declines to observations will be documented in the patients’ medical records by the co-researchers. Observers/co-researchers will not enter the rooms, be by the bedside or observe any staff providing care to patients who have declined. Before entering side rooms or approaching bedsides in bays, the clinician being observed will verbally check that the patient is happy for the researcher(s) to continue. This will constitute verbal consent. Written consent will not be obtained, as no other personally identifiable data are being collected, making it disproportionate to collect such data for a consent form.

###### Interviews

Patients, and where appropriate their personal consultees, will first be approached by a member of staff on the ward, most likely one of the co-researchers (excluding the THEO PD facilitator), who are considered a part of the direct care team. They will be informed about the study, the opportunity to participate in interviews and provided with an appropriate participant information sheet. Patients, and where appropriate their personal consultees, can then either contact the research team directly or inform ward staff of their interest in participating.

All participants (except those without capacity) will be required to complete a written consent form. For patients without capacity, their personal consultees will be involved in the interview and will need to provide consent for themselves as well as a declaration for the patient. In instances where personal consultees identify another relative or friend as more suitable for the interviews, that individual will be approached. It is recognised that a person’s clinical situation may change; hence, all activities with patients will be conducted by registered health professionals, ensuring that the assessments of capacity can continue even if consent was initially given during a period of capacity. If a patient loses capacity before data collection, a personal consultee will be approached.

All queries will be clarified by the co-researchers, and participants will be given sufficient time to consider their willingness to participate in the study, at least 24 hours, but realistically as long as they need. Translation services will be provided by the study team to facilitate the involvement of individuals with insufficient English language proficiency.

### Data collection

#### Values Clarification Exercise (all staff)

A VCE will be conducted at the start of the PD activities to create a shared vision and the ways of working among THEO co-researchers and ward staff. VCE helps develop a common purpose and vision, recognising different priorities and roles.^32^

Conducted in small groups to ensure that everyone interested has the opportunity to participate, VCE will be available to co-researchers and all ward staff within the first 3 months of the intervention period. Dates and times will be agreed on with ward staff to ensure that they are convenient and clinically appropriate. Staff participants will undertake this activity once, but co-researchers might participate multiple times as they assist in data collection. Each iteration allows co-researchers to influence the content and direction of the activity. It is anticipated that small groups will last between 1 and 3 hours, depending on group size, discussion level and other pressures on the day. If challenges arise, causing delays, the activity will be conducted at the earliest possible opportunity.

Participants will answer questions individually, then share their responses with the group. These will be collated, and themes identified. Through poster creation, synthesis will lead to a statement representing the ward’s purpose and its role in caring for older people. The THEO PD facilitator will assist all groups in navigating the VCE.

#### Leadership assessment (co-researchers only)

The Guiding Lights for Leadership, an appreciative 360° self-assessment tool,^33^ will be used at the start (within the first 3 months) and near the end (around month 9) of the THEO intervention. This tool aims to develop co-researchers into transformational and collective leaders and measure the leadership impact of the intervention. The self-assessment results will be used for personal and professional growth, shared and analysed collectively to guide future activities and may inform steps to enhance leadership potential for sustaining improvements beyond the study.

#### Workplace culture assessment (co-researchers only)

This exercise is for co-researchers to explore workplace culture at the beginning (within the first 3 months) and towards the end (around month 9) of the THEO intervention. Participants will choose their preferred culture assessment tool from the THEO PAR Handbook and work together to identify behaviours, patterns and rules. Sessions, facilitated by the THEO PD facilitator, may be held multiple times to ensure that all co-researchers can participate.

#### Staff and patient experience interviews using emotional touchpoints

The emotional touchpoint method asks participants to describe their experiences using a selection of emotional words.^34^ Participants will be asked to select words that best describe their feelings at key points in their care experience.

Open to all staff on the ward, co-researchers will use the emotional touchpoint approach to understand staff experiences on the participating ward. Co-researchers will interview other co-researchers and/or wider staff at between months 4 and 6 of the intervention to gather evidence on ward practices and identify gaps between ‘what we say’ and ‘what we do’ (supplemented by observations of care described in number 5 below). Three to six interviews with staff and up to three patients, and where appropriate their personal consultee, will be conducted, lasting up to 60 min each. Staff interviews will be recorded and transcribed, taking place at a mutually agreed time and location.

Patient interviews will be conducted face-to-face in a private location on the ward, audio recorded and transcribed verbatim. For potential patient participants who may not be well enough to mobilise, interviews will be conducted by their bedside with privacy ensured by drawing curtains. For patients with limited mental capacity, dyadic interviews with their personal consultee (or another suitable relative or friend identified by their personal consultee) will be conducted in a secure location within the hospital or by their bedsides, depending on their unique circumstances. If the interviewer observes any distress in patient participants, the interview will stop immediately and participants will be offered the opportunity to continue at a more convenient time, if desired.

#### Observations of care

The observation of care approach allows observers to understand the environmental and emotional context in which care takes place, explore the organisation and processes of care differently, celebrate successes and identify areas for improvement.^35^

Focusing on how care is delivered, co-researchers will work with the existing team members to observe care activities on participating wards, with observations scheduled based on co-researcher availability and ward pressures. Between one and three observations lasting 15–30 min will be conducted during the intervention. Two coresearchers, paired based on complementary skills, will perform the observations. Before staff undertake the activity, they will have an opportunity to talk through observing practice so that they understand the process and the principles of giving effective feedback.

This is a non-participatory activity for patients; so, they do not need to do anything differently than they would if the observations were not being conducted. Observers will respect patients’ autonomy and privacy and will leave the room if asked to do so. For patients with limited capacity, observers will watch out for facial and verbal cues of distress or disagreement to being in the room, and they would leave immediately if such cues were observed. Patients, and where appropriate their personal consultees, can verbally opt out at any time before or during the observations. However, it will not be possible to withdraw already collected observational data due to its anonymous nature. No personal data will be collected, and it will not be possible to identify participants from observational data, including notes. An observation sheet containing a few questions to prompt observations will be used.

On completing the observations, observers will have the opportunity to discuss their findings and determine how to provide feedback to the team. The observation of care approach will be used between months 4 and 6 to gather evidence on current practices in participating wards and help co-researchers identify gaps between ‘what we say’ and ‘what we do’.

#### Fourth generation evaluation (co-researchers only)

This exercise will be undertaken with co-researchers to cocreate action plans that will enable the THEO intervention registered nurses to find their place within the team and help the organisation determine the appropriate level of support required for their new roles. The fourth generation evaluation approach^36^ will consist of approximately monthly workshops (up to a maximum of 15), starting from month 1 and continuing until month 11. They might conclude earlier if all other activities are completed, but not before month 9. The workshops will last between 1 and 6 hours, depending on the size of the group, the level of discussion and other pressures on the day.

To ensure that all co-researchers are included, multiple workshops might be held to give everyone the opportunity to contribute. These workshops may be audio recorded to allow participants to listen back to the conversations and may also be transcribed verbatim. The need for this will be determined by the participants and whether they find it helpful.

#### Final participatory evaluation (co-researchers only)

The participatory evaluation^37^ is a collaborative and inclusive approach that actively engages all stakeholders in the evaluation process of a transformation.^25^ This activity will help conclude all the work conducted as a part of the facilitated PD activities. It should occur when participants are ready to incorporate their learning into everyday practice without facilitation or oversight. The timing will depend on the progress made by the ward, but it is anticipated that this will be in either month 10, 11 or 12 of the intervention period.

Participants will be encouraged to keep a journal. They will be asked to reflect on the content of this journal (for those who kept one) during this exercise. However, they will not provide the journal to the researchers. It is important that this journal be truly authentic, allowing participants to be honest about the intervention without worrying about others’ opinions.

Table 1 outlines the different data collection exercises, episodes, duration, number of rounds, data collection tools, participants and timelines for work package 1.

**Table 1:** Data collection exercises in work package 1

SN	Activity name	Description	Participants involved	Activity tool	Number of rounds	Activity duration	When in THEO lifecycle
1	Values Clarification Exercise	An activity aimed at creating a shared vision and explicit ways of working among everyone on the ward.	Staff working on the intervention ward	VCE tool	Once	Up to 3 hours per participant	First 3 months
			Co-researchers		Repeatedly to maximise participation		
2	Leadership assessment	A pre- and postintervention activity aimed at assessing and developing THEO co-researchers into transformational and collective leaders.	Co-researchers	Guiding lights for leadership tool through an appreciative 360° assessment	Two	Up to 3 hours per participant	First 3 months
							Month 9
3	Workplace culture assessment	A pre and post intervention activity aimed at exploring the workplace culture on the intervention ward.	Co-researchers	Bates teams culture tool or 15-step challenge	Two	Up to 3 hours per participant	First 3 months
							Month 9
4	Emotional touchpoint interviews	An activity exploring participants’ experiences on the ward, using a selection of emotional words.	Staff working on the intervention ward Patients on the ward		Once for 3–6 staff Once for 3–6 patients	Up to an hour	Between months 4 and 6
5	Observations of care	This exercise will involve observing care-related activities on the THEO intervention ward.	Staff working on the intervention ward	Predesigned observation sheet	Once but for 1–3 staff	Up to 20 min	Between months 4 and 6 or after the first three activities listed above.
6	Fourth generation evaluation	This activity will gather stakeholders’ perceptions of ongoing activity to ensure an iterative approach to THEO implementation activities.	Co-researchers		Up to 15	Up to a day depending on the depth of discussion	Monthly between months 1 and 11
7	Final participatory evaluation	A reflective exercise on key learnings, any transformative changes and recommendations for future implementation.	Co-researchers	Reflective model of choice	One	Up to a day depending on the depth of discussion	Between months 10 and 11, depending on when all other facilitated activities conclude.

THEO, therapeutic optimisation; VCE, values clarification exercise.

### Work package 2: quantitative data extraction

This work package will extract and aggregate quantitative data from the two participating NHS Trusts.

#### Objectives

The specific objectives of this work package are to determine the following.

1. Effectiveness and impact of the THEO intervention on specified quality indicators, such as length of stay and nurse-sensitive measures like falls and pressure ulcers, to mention a few.
2. Impact of the THEO intervention on other quality indicators, including but not limited to mortality, readmission rate, patient and staff satisfaction, staff recruitment and retention.

#### Eligibility criteria

There will be no active recruitment of participants for this work package. Only anonymised routinely collected data from in-patient admissions and staff records will be retrieved and included in the study analysis.

#### Data collection

Quantitative data will be sourced from routinely collected administrative data (patient and staff related) and will be extracted from 1 January 2015 to 30 days after the end of the intervention period, which lasts 12 months from the implementation start date. This will create two distinct periods: before (unexposed) and during (exposure period).

##### The unexposed (before THEO implementation) period: control

The unexposed period will span from 1 January 2015 up to the implementation start date for each NHS Trust. An additional 30 days of data (up to 30 days postimplementation start date) will be extracted to account for the readmissions of individuals admitted in the last month before the implementation start date. This period was chosen pragmatically to adjust for the impact of COVID-19 and the reconfiguration of NHS services during the pandemic. Data will be extracted at least 30 days after the intervention initiation but within 3 months.

##### The exposure (during THEO implementation) period: intervention

The exposure period will begin on the THEO implementation start date at each NHS Trust. Data will be extracted for at least 12 months from the implementation start date, plus an additional 30 days to account for readmissions of individuals exposed to the THEO intervention. These data will enable the evaluation of the THEO intervention’s impact over time.

### Data analysis

All statistical analyses will be performed using STATA and/or R software. Descriptive summaries (eg, number, proportions, means, SD and IQRs) and graphical plots will be presented for all collected data, categorised by data type and prespecified subgroups, including demographic data (eg, age, gender and deprivation).

#### Primary outcomes

The primary endpoint of the main study will be patient outcomes (eg, length of stay and nurse-sensitive measures). These endpoints will be analysed and reported separately by the study exposure period within the NHS Trusts.

#### Secondary outcomes

Secondary endpoints will include patient and staff-related outcomes such as mortality, readmission rates, patient satisfaction, retention rates and vacancy rates. Exploratory analysis will be conducted to identify associations between these outcomes and the THEO intervention.

### Work package 3: qualitative interviews

#### Objectives

Using semistructured interviews, this work package will explore the experiences of patients with mental capacity, patients who lack mental capacity (with their identified personal consultees) and staff members regarding the THEO intervention on older persons’ wards.

#### Eligibility criteria

Study participants will be recruited based on the eligibility criteria described below.

1. Members of staff within allied health professions (AHP), nursing and pharmacy staff assigned to and working/providing direct patient care on the wards where the study intervention is being delivered.
2. Patients who are admitted on a participating ward for at least 72 hours while the study intervention is being delivered.
3. For patients who are unable to consent for themselves, an identified personal consultee or relative/friend identified by the personal consultee and who has visited the patient while admitted to a participating ward will deliver the study intervention. The identified personal consultee does not have to be the legally recognised next of kin but:
  1. They must have had an active involvement with the patient during the intervention period, as determined by the clinical judgement of the nurses working on the ward.
  2. They must be confident in their ability to understand and respond to the patient, ensuring meaningful conversations.
  3. If personal consultees identify another relative or friend as more suitable for the interviews, that individual will be approached.

Translation services will be provided by the study team to facilitate the involvement of patients (and where applicable, their personal consultees) with insufficient English language proficiency.

#### Participant sampling and recruitment

Participants, including patients (both with and without mental capacity, along with their personal consultees, where applicable) and staff members, will be recruited through a purposive sampling technique. This will involve identifying and selecting individuals/groups that have experienced the implementation of the THEO intervention.

A generic notification email will be shared by the local-nominated collaborator to inform staff on the participating wards that they are working on a research ward and will be invited to participate in research activities 9 months into the intervention. Approximately, 8.5 months into the THEO intervention, study posters will be displayed at strategic locations within the participating wards. These posters will advise potential participants about the semistructured interviews and will contain QR codes linking to electronic participant information sheets and expression of interest forms. Participants can use these forms to signify their interest. The posters will also include contact details for the research team, allowing potential participants to ask any questions or seek clarifications. All queries will be addressed by the local collaborator or the qualitative researcher.

To recruit staff participants, an invitation email will be sent to staff members, advising them of the study purpose and their rights to participation and withdrawal. Staff can express their interest by completing the expression of interest form or by contacting the research team directly. All queries will be clarified by the researcher. Potential participants will then be sent a consent form to complete and will be given at least 24 hours to consider their participation. Verbal consent will be obtained from staff before commencing the interviews, which will be audio or video recorded depending on whether the interviews are conducted face-to-face or virtually.

To recruit patient participants, staff members will act as gatekeepers, informing patients and, where appropriate their personal consultees, about the study interviews. To maximise recruitment, the nominated local collaborator, considered a part of the direct care team, will visit the ward to speak to potential participants about the study. Participant information sheets will be shared with patients during the week before data collection, which will occur in the last 3 months of the intervention period. Patients interested in participating can complete an expression of interest form themselves or have the local collaborator do it on their behalf. The qualitative researcher will then contact potential participants to complete the consent form and arrange interviews.

To ascertain whether a patient is mentally capable to participate in the interviews, a clinician on the ward will assess the mental capacity of potential participants. This will help the local collaborator identify patients who can be contacted directly and those whose consultees need to be contacted with the study information. The mental capacity status of recruited participants will be documented in the patients’ medical notes.

Patients with capacity will complete a consent form and will have at least 24 hours to consider their participation. If they do not decide before discharge, they will not participate. Verbal consent will be obtained and audio recorded before interviews. For patients lacking capacity, they will receive a simplified information sheet, and their personal consultees will receive a consultee information sheet and a combined consent and declaration form. This form will capture the consultee’s consent and their declaration that the patient would want to participate. Verbal consent from both the patient and their consultee will be obtained and audio recorded before interviews. If a personal consultee identifies another relative or friend as more suitable for the interviews, that individual will be approached. Such individuals must be confident in their ability to understand and respond to the patients, ensuring that meaningful conversations are held. If no personal consultee or an alternative relative/friend is available, such patients will be excluded from the study.

#### Data collection

One-to-one, in-depth semistructured interviews will be conducted with up to 15 staff members and 15 patients per ward per Trust. This includes both patients with and without mental capacity. Patients lacking mental capacity will be recruited alongside their personal consultee or another suitable relative or friend for interviews. A dyadic interviewing technique^38^ will be used with their personal consultee or a suitable relative or friend who has had reasonable contact during the intervention. The total number of personal consultees or other relatives/friends will depend on the number of patients lacking mental capacity who participate in the dyadic interviews.

Appropriate interview guides, tailored to each participant group, will guide the discussions. For the dyadic interviews, a revised interview guide will be used to ensure that the data collected are comparable with one-to-one interviews. To gather more meaningful insights, the researcher will ask participants to focus on how they feel about the questions, rather than their recall.

For staff participants, interviews will be conducted either in-person within the hospital or online via Microsoft Teams, depending on logistics and staff preferences, and will last up to 60 min. Patient interviews will be conducted in-person within the hospital premises and will last up to 60 min but can be shorter if the participant finds this duration difficult. Interviews will be conducted at the patients’ bedsides. To ensure privacy for participants in multioccupancy rooms, curtains will be drawn unless there is a private space on the ward where patients can be relocated for the duration of the interview. This approach aligns with the privacy standards for discussing health-related information protected under the Common Law Duty of Confidentiality and is deemed appropriate for this context. Moving patients off the ward is not suitable for the majority of this patient population. For patients in private rooms, interviews will be held there.

Interviews will take place at a time and location agreed on based on the participants’ preferences.

#### Data analysis

Qualitative data will be transcribed verbatim and analysed using NVivo, a qualitative data analysis software. The transcribed data will be thematically analysed using the six-step reflexive approach.^39^ Thematic analysis will be conducted independently by two members of the research team, who will then come together to agree on key themes and resolve any differences, thereby enhancing rigour.

### Work package 4: mixed methods process evaluation

This work package will evaluate the process of implementing and delivering the THEO intervention. Process evaluations help researchers understand why an intervention succeeded or failed by examining its implementation, contextual factors and providing insights for interpreting the results.^40^

#### Objectives

The overarching aim of this process evaluation is to test the initial programme theory by examining the contexts and mechanisms of the THEO intervention in two NHS Trusts, exploring both positive and negative contextual factors and using quantitative and qualitative methods to inform a refined programme theory for future implementation.

#### Design

A mixed methods research approach, using both qualitative and quantitative data collection techniques.

#### Initial programme theory

**Figure 1:**
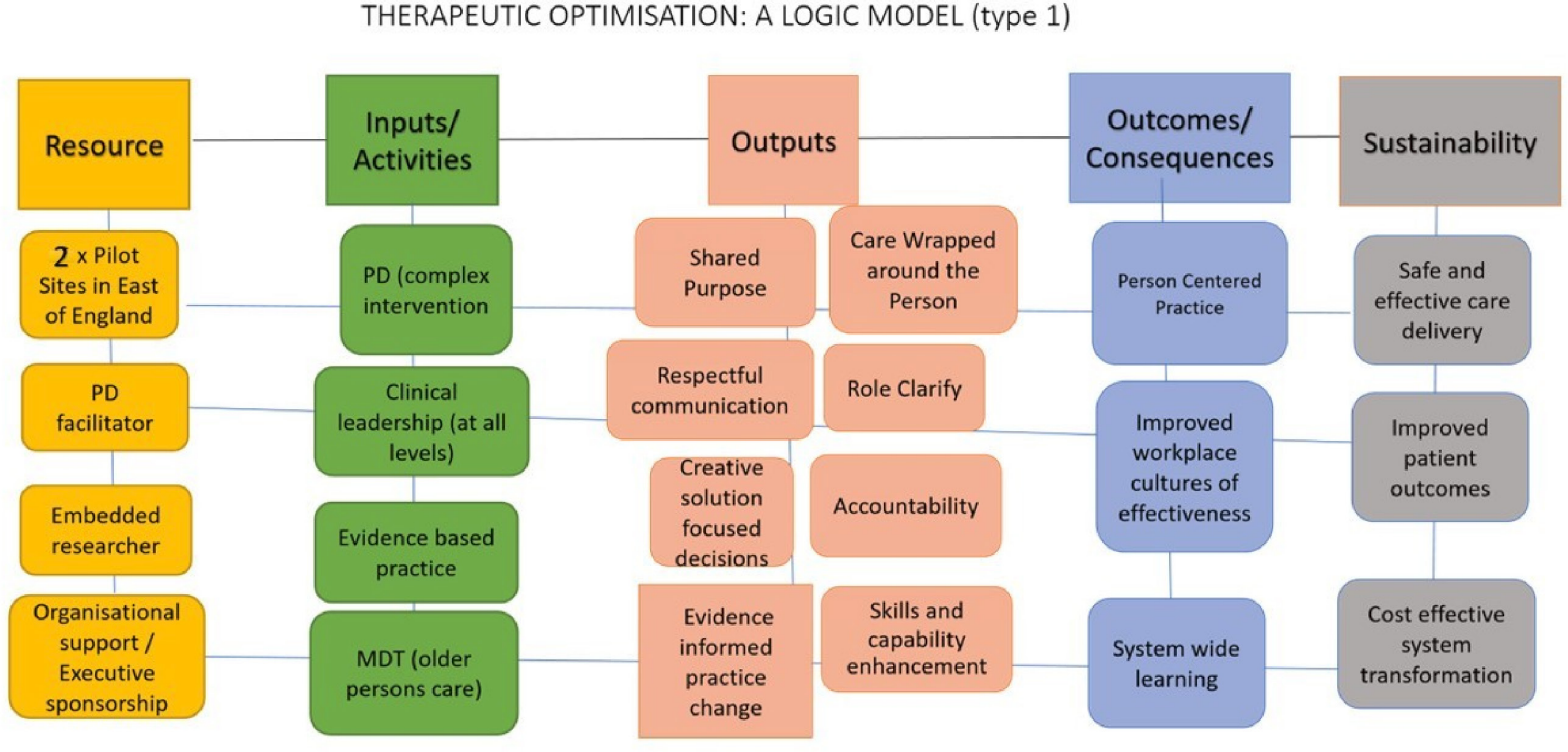
THEO intervention logic model. MDT, multidisciplinary; PD, practice development; THEO, therapeutic optimisation.

A realist programme theory specifies the outcomes linked to the intervention, the mechanisms generating these outcomes and the contextual features affecting them. An initial logic model (see figure 1) was designed to represent that how contextual factors may impact the implementation inputs, including THEO components and strategies. This model will inform the outcome measures used to evaluate the study. Understanding this logic is crucial for comprehending the THEO intervention and building knowledge on how and why it is expected to work in the participating trusts.^41^

#### Study participants

Participants will include two broad staff groups: co-researchers and staff members involved in direct patient care on the intervention wards who are not a part of the coresearcher team.

#### Qualitative process evaluation

The qualitative process evaluation will follow a coproduction approach, inviting key stakeholders to participate in guided discussion workshops after the THEO intervention has been in place for 9 months or during the last 3 months of the intervention period, whichever is longer. A coproduction team will identify and evaluate the contextual factors that influenced the THEO intervention’s implementation, interpret the findings for each stakeholder and collaboratively share or apply these insights for future interventions.^42^ This work aims to provide insights into the systems, culture and circumstances, which impacted the overall reported effectiveness of the THEO intervention.

##### Participant sampling and recruitment

A nominated local collaborator will act as a gatekeeper and will email key stakeholders in each participating NHS Trust to invite them to the workshops. Potential participants can express interest by completing an expression of interest form or directly contacting the local collaborator or research team. Participant information sheet detailing the study purpose and participants’ rights will be included in the email. The qualitative researcher will address all queries.

Participants will be recruited through purposive sampling, including key stakeholders involved in implementing and delivering the THEO intervention in the two NHS Trusts. Posters will also be displayed on the wards to actively encourage staff participation.

Participation will be voluntary, and potential participants will be given at least 24 hours to consider participation and complete an electronic consent form. Verbal consent will be obtained and recorded on the workshop day. It is expected that all co-researchers and ward managers on the older persons’ wards will participate, but if any ward manager declines, their deputies will be invited. Members of staff who do not belong to the coresearcher group will be recruited based on maximum variation to ensure sample heterogeneity.^43^

##### Procedure and data collection

Using a coproduction approach, four workshop sessions will be conducted with key stakeholders across the two participating NHS Trusts. Each NHS Trust will host two workshops, each with approximately five participants, totalling ten staff members per Trust. A discussion guide underpinned by the RE framework will guide the discussions to gain insights into the contextual factors, barriers and facilitators influencing the THEO intervention’s implementation. Workshops will be face-to-face, facilitated by a research team member and last about 3 hours. Participants will receive study information, and all views will be respected, managing power dynamics and interactions. Ethical guidelines will be followed, including obtaining verbal consent to record and take notes. Participants are free to withdraw at any stage of the workshops without their rights being affected. However, due to the interactive and interdependent nature of the transcription, participants are unable to withdraw any previously given data within the discussions.

Additionally, the THEO PD facilitator and the THEO intervention registered nurses will complete engagement logs each time they engage with the ward for study-specific activities. These logs will include standard questions to capture the details of the implementation process and any changes within each Trust that might affect the intervention’s success. Completing these logs is mandatory for this group of participants. Information from these logs will form a part of the process evaluation data to address the work package objectives.

##### Data analysis

Data will be transferred into NVivo and thematically analysed. Thematic analysis will be independently conducted by two members of the research team coming together to agree on key themes and resolve any differences, thereby enhancing rigour. A deductive data analysis approach will be undertaken, using the CMO configuration to identify mechanisms (how intervention components result in changes) and contextual factors (conditions influencing these mechanisms) associated with outcome variations.^27^

### Quantitative process evaluation

This aspect of the work package will use routinely and prospectively collected staff data to evaluate the feasibility and fidelity of the implementation of the THEO intervention.

#### Participant sampling and recruitment

Study questionnaires will be sent to all staff involved in the implementation and delivery of the THEO intervention. The nominated local collaborator and communication team within each NHS Trust will be responsible for distributing the questionnaires to the appropriate participants via NHS email. The research team will coordinate with local collaborators to ensure timely email distribution. Participants will receive sufficient information to make an informed decision, and completing the questionnaire will imply consent for their data to be used in the study, as specified on the questionnaire’s information page. To ensure anonymous data collection, questionnaires will be administered online using Qualtrics software. Once the survey is submitted, it will not be possible to withdraw data as they will be anonymous.

#### Data collection

The normalisation measure development (NoMAD) questionnaire,^44^ a validated tool, will be administered. Surveys will be administered to staff on the participating wards at 3- and 12-month poststart date.

To assess staff awareness of the THEO intervention and its intentions (reach), a questionnaire developed by the research team will be circulated to staff across each participating NHS Trust. The questionnaire will include closed questions to assess awareness of the THEO intervention and its intentions. This questionnaire will be administered at 9-month poststart date.

Both questionnaires will be administered through the support of the nominated local collaborator, strategic leads and communications teams in each participating Trust. Potential participants will have up to 4 weeks to read the information and complete the questionnaires. One reminder email will be sent 2 weeks after the initial email. Survey completion is voluntary; however, due to the anonymous nature of the collected data, they cannot be withdrawn once submitted.

#### Data analysis

Statistical analysis will be performed using STATA and/or R software. Standard descriptive summaries (numbers, proportions, means, SD and IQRs) and graphical plots will be presented for all collected data based on the data type.

### Ensuring rigour in the qualitative components

To enhance the trustworthiness of the qualitative components, we will employ multiple strategies consistent with Lincoln and Guba’s evaluative criteria.^45^ Credibility will be supported through the triangulation of data from diverse sources (eg, staff, patients and observations), peer debriefing within the research team and iterative member checking conducted during the co-production workshops. Within the workshops, co-researchers will undertake the theming and analysis of all gathered evidence, ensuring that the process is cross checked by all team members. The THEO PD facilitator will be present to challenge any assumptions made by the team, ensuring a critical examination of the data. Co-researchers, who come from various roles within the ward team, will bring a range of perspectives, further enhancing the validity of the findings. In addition, the THEO intervention registered nurses, although embedded within the NHS Trusts, are new to the ward and bring a fresh set of eyes, offering an element of independence that is valuable both from a trustworthiness standpoint and in terms of developing relationships that are essential for the success of the intervention.

Dependability will be ensured by maintaining a comprehensive audit trail that documents methodological decisions, alongside detailed records of facilitation processes using the THEO PAR Handbook. These strategies will ensure that the research process remains transparent and methodologically consistent.

Reflexivity will be systematically embedded through scheduled reflexive discussions involving both the research team and co-researchers, aimed at critically examining the influence of our positionalities, assumptions and potential biases. Co-researchers will be encouraged to maintain reflective journals throughout the intervention period. Where appropriate and with informed consent, selected excerpts from these journals will be incorporated to enrich data interpretation and facilitate critical engagement with the evolving context of implementation.

### Ensuring rigour in the quantitative components

To guarantee the trustworthiness of the quantitative component, we will implement several rigorous quality assurance procedures.

- Data validation will be performed through systematic cleaning of all extracted datasets, including cross checks for completeness, internal consistency and formatting. Any discrepancies identified will be resolved collaboratively with the data-providing NHS Trusts using established data dictionaries.
- Triangulation will be applied by comparing and interpreting quantitative trends (such as staff retention and length of stay) alongside qualitative data derived from interviews, observations and coproduction workshops, consistent with our convergent mixed methods design.
- Reliability will be assessed through consistency testing of key variables across the two study sites and between pre- and postintervention phases, supplemented by sensitivity analyses where appropriate.
- The use of validated instruments, such as the NoMAD questionnaire, will further ensure the reliability and comparability of implementation data across multiple timepoints and locations.

Collectively, these measures will strengthen the internal validity and enhance the generalisability of the quantitative findings.

### Data management

For all qualitative data, audio and video recordings will be transcribed verbatim, promptly and then securely destroyed after verification. A specialist third-party company may handle transcription, ensuring compliance with data protection laws. Electronic data will be securely stored on password-protected, encrypted laptops owned by the University of East Anglia (for work package 1) and the University of Staffordshire (for work packages 3 and 4). Personally identifiable data will be stored separately from anonymised research data for 3 years poststudy and then securely destroyed. Consent forms will be stored digitally on Microsoft Teams.

For work package 2, quantitative data from the hospital patient administration system will be anonymised on a secure NHS Trust computer. The NHS Trust can transfer data using its preferred method or a secure facility provided by the University of Staffordshire. Data will be stored on the University of Staffordshire’s cloud storage. Patient hospital numbers will be anonymised using an algorithm applied by hospital staff. Research team members will only access anonymised data. Patients’ ages will be reported in years, not dates of birth. Surveys will be administered online using Qualtrics software. Additional process delivery data from NHS Trusts will be anonymised before sharing with the University of Staffordshire.

Data management will comply with UK General Data Protection Regulation (GDPR, 2018) and the Data Protection Act (2018). Only the study team will have access to the data. Anonymised data may be retained indefinitely, in line with open access requirements, but for at least 10 years poststudy completion.

### Ethics and dissemination

The study protocol has undergone the University of Staffordshire Independent Peer Review process and Health Research Authority (IRAS ID: 334473) approval and Research Ethics Committee favourable opinion.

### Ethical considerations

Standardised ethical guidelines will be followed throughout the study. All potential participants will be approached in a manner that respects their privacy and data protection rights. The research team will not receive any personally identifiable data without prior consent. Each participating NHS Trust will act as a gatekeeper, promoting and disseminating study information to staff, patients and, where applicable, their personal and/or professional consultees for patients with limited mental capacity.

Informed consent will be sought for all data collected in work packages 1, 3 and 4, with data coded anonymously and stored separately from personally identifiable information. Potential participants will receive a participant information sheet and have time to consider participation before completing a consent form. Verbal consent will be recorded on the interview day.

Participation in work packages 1, 3 and 4 is entirely voluntary, with no impact on careers, entitlements, care or support for those who decline. Participants can withdraw during data collection (one-to-one or dyadic interviews) without explanation. However, due to the nature of group discussions in work packages 1 and 4, participants cannot withdraw their data once provided, which will be clarified in the participant information sheet. For one-to-one interviews, if the recording has not been deleted (ie, transcription is ongoing), it will be deleted on withdrawal. Workshop discussion data cannot be deleted as it would affect the remaining participants’ data. Quantitative data will be collected via online surveys, with completion implying consent. Interviews and workshops are not expected to cause distress, but support will be provided if needed.

Data collected will remain confidential, with participants reminded that any safeguarding issues or criminal activities disclosed will be reported to appropriate personnel. In work package 2, no personally identifiable data will be shared with the research team, maintaining confidentiality.

### Dissemination

Study findings will be shared with key stakeholders, including the participating NHS Trusts. The results will be published in high-impact, peer-reviewed journals and presented at relevant conferences.

## Supplementary material

10.1136/bmjopen-2025-102529 online supplemental file 1
