# Supplementary material for: Quasi-experimental study on the effectiveness and impact of implementing nurse-led ‘therapeutic optimisation’ (THEO) intervention in two older persons wards: a mixed methods study protocol
Source: BMJ Open. 2025 Aug 26;15(8):e102529. doi: 10.1136/bmjopen-2025-102529 (PMC12382520; doi:10.1136/bmjopen-2025-102529)
Supplement: online supplemental file 1 [file bmjopen-15-8-s001.docx]

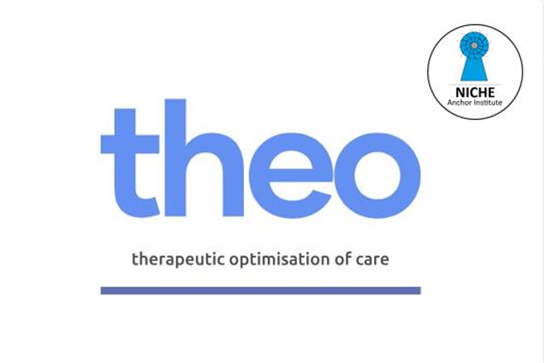


**THEO Participatory Action Research Handbook**

**Implementing and capturing evidence of practice development through a participatory process of Therapeutic Optimisation (THEO)**

*“The right staff, providing the right care (to the person/patient), to optimise care outcomes.”*

# Table of Contents

[Table of Contents 2](#_Toc174977261)

[Working Together to promote active participation within the Therapeutic Optimisation (THEO) study 5](#_Toc174977262)

[How to use this handbook 5](#_Toc174977263)

[Where to start 6](#_Toc174977264)

[Section 1: What is Participatory Action Research? 6](#_Toc174977265)

[1.1. Introduction to PAR 6](#_Toc174977266)

[1.2. Principles of Engagement in PAR 7](#_Toc174977267)

[1.3. Who will be involved in THEO? 9](#_Toc174977268)

[1.4. Ethical Considerations 10](#_Toc174977269)

[PAR Section References 11](#_Toc174977270)

[Section 2: Practice Development Activities 12](#_Toc174977271)

[References for Practice Development 13](#_Toc174977272)

[2.1. Values Clarification Exercise 13](#_Toc174977273)

[2.1.1. Introduction 13](#_Toc174977274)

[2.1.2. What does a values clarification exercise look like? 14](#_Toc174977275)

[2.1.3. Who will be involved? 14](#_Toc174977276)

[2.1.4. How will the VCE be used? 15](#_Toc174977277)

[2.1.5. What happens to the information arising from the values clarification exercise? 15](#_Toc174977278)

[2.1.6. VCE References 15](#_Toc174977279)

[2.2. Pre and post leadership assessment for individual participants 16](#_Toc174977280)

[2.2.1. Introduction 16](#_Toc174977281)

[2.2.2. How the leadership self-assessment tool will be used? 17](#_Toc174977282)

[2.2.3. Who will be involved and when will it be used? 17](#_Toc174977283)

[2.2.4. The leadership self-assessment tool 17](#_Toc174977284)

[Leadership Self-Assessment References 19](#_Toc174977285)

[2.3. Pre and Post Workplace Culture Assessment 20](#_Toc174977286)

[2.3.1. Introduction 20](#_Toc174977287)

[2.3.2. How the workplace culture assessments will be used and who will be involved? 21](#_Toc174977288)

[2.3.3. Workplace Culture Assessment activities 21](#_Toc174977289)

[Workplace Culture Assessment References 23](#_Toc174977290)

[2.4. Fourth Generation Evaluation – Claims, concerns and issues exercise (Guba and Lincoln, 1989) 24](#_Toc174977291)

[2.4.1. Introduction 24](#_Toc174977292)

[2.4.2. How and when the CCI will be used and who will be involved ? 25](#_Toc174977293)

[2.4.3. The CCI activity 26](#_Toc174977294)

[CCI References 26](#_Toc174977295)

[2.5. Emotional Touch point Interviews 26](#_Toc174977296)

[2.5.1. Introduction 27](#_Toc174977297)

[2.5.2. Emotional Touchpoints websites (free access) 27](#_Toc174977298)

[2.5.3. How will the approach be used? 27](#_Toc174977299)

[Emotional Touchpoints References 28](#_Toc174977300)

[2.6. Observation of Care – Workplace observations 28](#_Toc174977301)

[2.6.1. Introduction 29](#_Toc174977302)

[2.6.2. How and when will the Observation of Care approach be used? 29](#_Toc174977303)

[2.6.3. Example of the Observation of Care tool for recording observation 30](#_Toc174977304)

[2.6.4. Observation of practice feedback template 31](#_Toc174977305)

[2.6.5. Principles of Giving and Receiving Feedback 32](#_Toc174977306)

[2.6.6. Observation of Care: Frequently Asked Questions 33](#_Toc174977307)

[Observation of Care References 34](#_Toc174977308)

[2.7. Final Participatory Evaluation 34](#_Toc174977309)

[2.7.1. Introduction 34](#_Toc174977310)

[2.7.2. How and when will the evaluation be used? 35](#_Toc174977311)

[2.7.3. A series of reflective questions for individual use 35](#_Toc174977312)

[2.7.4. Keeping a reflective journal 35](#_Toc174977313)

[Final Participatory Evaluation References 36](#_Toc174977314)

[2.8. Shared Learning Events 36](#_Toc174977315)

[2.8.1. Introduction 36](#_Toc174977316)

[2.8.2. When will this happen? 36](#_Toc174977317)

[2.8.3. Who and what will be involved? 36](#_Toc174977318)

[Section 3: Closing the intervention phase of THEO 36](#_Toc174977319)

[3.1. Lessons Learned 36](#_Toc174977320)

[Appendix 1: Table showing the THEO implementation activities and when they will occur during implementation of the THEO intervention 38](#_Toc174977321)

[Appendix 2 : Preliminary theory of change/logic model and associated PD evidence source 39](#_Toc174977322)

# Working Together to promote active participation within the Therapeutic Optimisation (THEO) study

The THEO participatory action research (PAR) handbook has been devised as a resource for facilitating active engagement and participation with the intervention phase of the THEO study. This handbook is particularly relevant to the co-researcher team who will be facilitating the implementation of this intervention. There are different sections that outline the activities that as participants, we will work together to achieve the right care for patients, delivered safely, and at the right time, to optimise care outcomes and the therapeutic care environment.

These approaches are frequently used in Practice Development (Manley et al., 2021) projects, based on decades of experience of working with clinical teams to promote a workplace culture that seeks to continuously improve care experience. The premise is how to use the workplace as the basis of learning to enhance the daily routines and practices, based on best evidence available to continuously focus activities on the person, seeking to provide safe, evidence-based practices that contribute to co-creating a workplace culture of effectiveness.

## How to use this handbook

This handbook is intended to be the main source of the activities that will be used within the implementation of the THEO intervention. Section 1 of the handbook will tell you what participatory action research (PAR) is and its principles of engagement. Section 2 will introduce the different PAR activities that will inform the implementation of the THEO intervention. This will include the purpose of each activity, the different assessment tools (where relevant) and how these will be used, who will be involved and the relevant references for each activity. Section 3 will conclude the handbook with information regarding closing the implementation phase of the THEO intervention.

You should have already reviewed the researcher and staff information and consent booklet which summarises these activities. You should also have provided your informed consent to participate in some or all of these activities prior to receiving this THEO intervention handbook . You will work with the THEO practice development (PD) Facilitator, who will guide you through implementing and participating in these activities, which will all be influenced by your daily practice and work experiences. Exploring what is working well, where and how improvements can be made, will be undertaken as a shared activity and systematic process of inquiry. Considering each aspect of the care environment, these different activities will be a process of active exploration (along with inviting wider participation as part of the process) of what, why and how practice can be enhanced. The evidence gathered, through these activities will enable the co-researcher team to gain new and valuable insights, information and ideas for where and how changes can be achieved, focusing on improving the care experience for the patients who receive care, people we work with, and those who will benefit from improvements.

## Where to start

Let us begin this process by looking at how you will be working as active participants in the research, then move to explore the activities you will be using to gather evidence and share learning along the way. We invite you to participate with us on this practice development journey so that our learning is continuously being used as a process of discovery (enlightenment), critically informed action (empowerment), innovation and sustainable change (transformation) as the THEO study progresses.

# Section 1: What is Participatory Action Research?

This first section explores participatory action research (PAR) and the key principles of engagement.

We anticipate it will take about 20 minutes to read through this section. However, each aspect will be discussed during the introductory phase of implementing the THEO intervention as a process of learning, negotiation and clarification of what is expected as an active participant.

## Introduction to PAR

Lloyd-Evans et al. (2023) describes how participatory action research (PAR) attempts to move away from independently observing, identifying and theorising about problems of and on ‘others’ but instead, works to engage communities in the process of inquiry, by bringing their knowledge of situations into any investigation of social action and interactions (Kindon, Pain and Kesby, 2007; Askins and Pain, 2011; Lloyd-Evans, 2016; Askins, 2018). Based on ideas of shared, or equalling the power relationships through working in collaboration, as a process of community action, PAR focuses on the notion that communities themselves have the knowledge, skills and expertise to best understand local needs and how to address changes through their lived experiences. PAR seeks to disrupt more traditional hierarchical power relations often seen between researchers and those who they study (the researched) by locating knowledge generation at the local level and enabling research participants (or communities) to explore and address issues that matter most to them.

The use of participatory methods helps break down barriers between communities and services providers and it is this community-centred approach that creates and strengthens the relationships and trust that are foundational to lasting social change. Russell (2021) discusses that PAR unfolds as a process of negotiation with research partners, focused on gathering response to the problems and needs of those partners. This process of negotiation gives more control to the people who are living the experience and their engagement with pinpointing problems and finding solutions, which helps to ensure that projects and their impact are relevant and sustainable into the long term. Participatory methodologies are becoming increasingly popular in addressing social justice issues as they attempt to democratise the research process, bring new voices to the table and challenge traditional, hierarchical notions of power.

Community-based participatory research (CBPR), often used interchangeably as participatory research and participatory action research refers to a collaborative study in which people whose lives might be affected by the issues being researched are equal partners in the processes of designing, undertaking and disseminating research. The aim is to effectively influence change, because those at the heart of the issue are developing the solutions together, as a democratic process and therefore more likely to be meaningful and sustainable. CBPR can be regarded as a specific type of ‘co-produced’ or ‘co-created’ research, in which the research partners include community members (Centre for Social Justice and Community Action & National Coordinating Centre for Public Engagement, 2022).

## Principles of Engagement in PAR

As THEO will be based within busy clinical/workplace settings where things change constantly, an interactive engagement framework will also be followed to guide the process of engaging in the activities that inform the implementation of the THEO intervention.

The table 1 includes these principles of engagement which are informed by the Centre for Social Justice and Community Action & National Coordinating Centre for Public Engagement (2022). The principles outlined will be used throughout the study period as a way of checking in with participants, and as agreed ways of working from which to discuss, clarify, and agree on ways of working, at the commencement of the study, then returned to again at different points of the study period, as aspects of the study influence new insights and approaches.

Table 1: THEO Key Principles of Engagement

| **THEO Principles of Engagement** |
| --- |
| 1**.Mutual respect**: developing research relationships based on mutual respect, including a commitment to:  • agreeing what counts as mutual respect in particular contexts.  • everyone involved being prepared to listen to the voices of others.  • accepting that people have diverse perspectives, different forms of expertise and ways of knowing that may be equally valuable in the research process. |
| 2. **Equity and inclusion**: encouraging and enabling people from a range of backgrounds and identities (e.g., ethnicity, faith, class, education, gender, sexual orientation, (dis)ability, age) to lead, design and take part in the research, including a commitment to:  • seeking actively to include people whose voices are often ignored.  • challenging discriminatory and oppressive attitudes and behaviours.  • ensuring information, venues and formats for meetings are accessible to all. |
| 3. **Democratic participation**: encouraging and enabling those involved in the research to contribute meaningfully to decision-making and other aspects of the research process according to skill, interest and collective need, including a commitment to:  • acknowledging and discussing differences in the status and power of people involved in the research and working towards sharing power more equally.  • communicating in language everyone can understand, including arranging translation or interpretation if required.  • using participatory research methods that build on, share and develop different skills and expertise. |
| 4. **Active learning**: seeing research collaboration and the process of research as providing opportunities to learn from each other, including a commitment to:  • ensuring there is time to identify and reflect on learning during the research, and on ways people learn, both together and individually.  • offering all those involved the chance to learn from each other and share their learning with wider audiences.  • sharing responsibility for interpreting the research findings and their implications for practice. |
| 5. **Making a difference**: promoting research that creates positive change for communities of place, interest or identity, including by:  • engaging in debates about what counts as ‘positive’ change, including broader environmental sustainability as well as human needs or spiritual development, and being open to the possibility of not knowing in advance what making a ‘positive difference’ might mean.  • valuing the learning and other benefits for individuals and groups from the research process as well as the outputs and outcomes of the research.  • building a goal of positive change into every stage of the research. |
| 6. **Collective action**: individuals and groups working together to achieve change through the research, including a commitment to:  • identifying common and complementary goals that meet partners’ differing needs for the research.  • working for agreed visions of how to share knowledge and power more equitably and promote social change and social justice.  • recognising and working with conflicting rights and interests expressed by different interest groups, communities of practice or place. |
| 7. **Personal integrity**: people conducting the research behaving reliably, honestly and in a transparent and trustworthy fashion, including a commitment to:  • working within the principles of CBPR.  • ensuring accurate and honest analysis and reporting of research.  • being open to challenge and change, recognising and reflecting on one’s own privileges and prejudices and being flexible and prepared to work with conflict. |

## Who will be involved in THEO?

Some people are involved in THEO because of their job. Some people are being invited to participate because they work or are cared for on the ward where the THEO intervention is taking place. We have broken the participants into three groups: co-researchers, staff, and patients and their consultees. For the purpose of this study, a personal consultee is defined as a person who has had reasonable contact with the patients whilst the THEO intervention is in place. This may include carer, family, and friends (Please see Appendix 1 for who will be involved in the different activities)

1. Co-researchers comprise:

- The THEO Practice Development Facilitator (lead intervention researcher and a staff member from University of East Anglia with previous experience of leading practice development projects nationally). This is Joanne Odell, and she is involved by the nature of her job.
- The THEO intervention (band 6 and 7) registered nurses (embedded researchers). These roles exist explicitly for the purposes of delivering the THEO intervention and therefore their participation is part of their job.
- Self-selected or consensus nominated (using a shared governance model) clinical representation from the units multidisciplinary (MDT) team who will act as co-researchers. It is anticipated 6-8 people will be recruited to participate.

1. Staff participants are members of the workforce who are not acting as co-researchers but who work on the ward where the THEO intervention is being delivered. The co-researchers will invite wider participation from the MDT team for various activities. There will also be occasions when staff participants from the organisation will also be invited to contribute via specific activities.
2. Patients who are being cared for on the ward and their consultees (when a patient lacks mental capacity and is unable to advocate for themselves). The co-researchers will invite participation for specific activities.

## Ethical Considerations

You will have already reviewed the researcher and staff information and consent booklet which incorporates the study purpose, the activities that will be undertaken as part of PAR, ethical considerations for this study including your rights to participation and withdrawal, how we will manage your data and the contact details of the research team. Similarly, you will have already provided your informed consent to participate in some or all of the activities that will be undertaken as part of implementing the THEO intervention, but we will check in with you that you are still happy to take part in these activities on the day the activities are due to occur. In addition, at each stage of the PAR, ways of working will be negotiated and co-created between the co-researchers and other participants to take into consideration clinical priorities and contextual circumstances (Kavanaugh & Ayres, 1998). The following sections highlight your ethical considerations expected from your interactions with staff participants, patients, and their consultees, where applicable.

The staff information and consent booklet will be shared with all potential staff participants – the same one that you received. This incorporates study information including purpose, the different activities taking place as part of implementing the THEO intervention, rights to participation and withdrawal, and how we will use any information provided during the activities and contact details of the research team. Staff participants will also be able to complete the consent form in this booklet, to indicate the aspects of the study they want to participate in. Similarly for patients, and where applicable, their consultees, they will be provided with relevant participant information sheet for the different aspects they can participate in and will be required to complete a consent form to indicate their willingness to take part in the study. For patients lacking mental capacity, their consultees will provide a declaration that they would want to participate in the study activities, and where joint patient and consultee participation is required, consultees will complete their own consent form. Their participation is entirely voluntary, and participants should be given at least 24 hours to consider their participation in the study activities. Informed consent must be sought from all participants prior to taking part in the activities and consent should be verbally checked before specific study activities take place.

You are required to approach participants in the THEO implementation activities in a way that does not breach their right to privacy and data protection. However, if potential participants should decline to participate or opt-out of any aspect of the THEO activities whilst it is ongoing, there will be no impact on their careers, entitlements (such as study and annual leave), care or support. Participants are not required to provide an explanation of why they decline to participate or opt-out of any aspect of the THEO activities.

Participants’ identity and all data obtained from them will be kept confidentially. These data will be coded anonymously. Unless where safeguarding concerns arise, the co-researchers will not divulge the details of participants to anyone outside of the research team. Documents that include personally identifiable information will be stored separately to the coded research data and only the research team will be able to make the link between the two. We will not publish or share data/information that would compromise participants’ identity.

## PAR Section References

Askins, K. and Pain, R. (2011) ‘Contact Zones: Participation, materiality and the messiness of interaction’, Environment and Planning D: Society and Space 29(5): 803-821

Askins, K. (2018) ‘Feminist Geographies and Participatory Action Research: Co-producing narratives with people and place’, Gender Place and Culture: A Journal of Feminist Geography 25(1): 1-18.

Centre for Social Justice and Community Action & National Coordinating Centre for Public Engagement (2022) Community-based participatory research: A guide to ethical principles and practice (2nd edition), <https://durham-repository.worktribe.com/preview/2288466/2288453VoR.pdf>

Kavanaugh, K., & Ayres, L. (1998). “Not as bad as it could have been”: Assessing and mitigating harm during research interviews on sensitive topics. *Research in nursing & health*, *21*(1), 91-97.

Kindon, S., Pain, R. and Kesby, M. (eds) (2007) Participatory Action Research Approaches and Methods: Connecting people, participation and place, New York: Routledge.

Lloyd-Evans, S. (2016) ‘Focus Groups, Community Engagement and Researching with Young People’, in: Evans, R., Holt, L. and Skelton, T. (eds) Methodological Approaches: Geographies of children and young people, vol 2. Springer: Singapore.

Lloyd-Evans, S., Oenga, E., Zischka, L., Mpofu-Coles, A., Woronka, R., Oveson, M., Hookway, D., Cleaver, M., Duval, S., Karanja, E. and Gomma, T., (2023). Participatory Action Research: a toolkit. [Participatory Action Research: A Toolkit (reading.ac.uk)](https://research.reading.ac.uk/community-based-research/wp-content/uploads/sites/114/2023/06/PAR-Toolkit-v10.pdf)

Manley, K. Wilson, V. and Øye (2021) (Eds) *International practice development in health and social care*. Wiley Blackwell

Russell, K (2021) Participatory Action research: Research Ethics Guidance Notes. University of East Anglia [University Of East Anglia – Research Ethics Guidance Note (Uea.Ac.Uk)](https://my.uea.ac.uk/documents/20142/15431415/09.+Participatory+Research+-+FINAL+01+08+21.pdf/0f02917e-0a54-e3e1-f75a-91279611d551?t=1628157609746)

# Section 2: Practice Development Activities

Garbett and McCormack (2002) describe that Practice Development (PD) is focussed on the culture and context where the intervention occurs, the translation of research findings and evidence into practice, a clarification of the similarities and differences between PD and professional development, the use of an 'action research' approach, consumer involvement and consultation, person-centred care, reflection on and in practice, leadership and facilitation. Since this publication, the theory and concept of practice development has been refined as more has been learnt about this way of working. Manley, Wilson and Oye, (2021) argue that there is a collection of evidence, that demonstrates the impact of PD on people, their transformation, their teams, users’ experiences but argue that its potential to influence systems more widely is still underutilised. They describe how managerial cultures or single top-down methodology approaches often ignore the importance of people or fail to use practitioners’ expertise as a source of social capital. Manley, Wilson and Oye (2021, p7) argue that the emphasis for Practice Development (PD) remains on person-centred care, cultures and systems as well as working with complexity and research practice “with people” rather than “on” people.

In this section of the handbook, the background to the different activities that will be used to gather evidence within a participatory action research (PAR) approach is provided. Each activity is introduced in a separate heading below and can be explored as standalone or in conjunction with other activities. An overview of all the activities, who will be involved and the timings of these within THEO can be found in Appendix 1. The combination of these activities has been identified as a complex intervention. (Please see evidence table in Appendix 2).

## References for Practice Development

Garbett, R. and McCormack, B. (2002) A concept analysis of practice development. *NT Research*. Vol. 7. No. 2. pp 87-100.

Manley, K. Wilson, V. Oye, C. (Editors) (2021) *International Practice Development in Health and Social* Wiley Blackwell.

## 2.1. Values Clarification Exercise

This section explores the process of using our values and beliefs to create a shared purpose. We anticipate this section will take 10-20 minutes to read. However, you will be facilitated to use the approach by the THEO PD facilitator as part of the process of evidence capture for THEO in your role as a co-researcher.

### 2.1.1. Introduction

A values clarification exercise (Warfield & Manley 1990; Manley 1992) (VCE) is a tool frequently used within practice development for developing a common shared vision and purpose at the start of a project or as a process for bringing teams or stakeholders together to create a common purpose. It can be used for developing a shared vision about the agreed areas of investigation or improvements. It is a process that also is sensitive to recognising different priorities, whether arising from participants, the development of role definitions, competency levels, achieving effective team working and developing agreed strategic direction.

VCE is often the starting point for cultural change, as values and beliefs influence behaviour. Through making explicit values and beliefs at the commencement of a project, or activity schedule, participants are taking the first steps to making their contribution a reality, and as a mutually informed and agreed approach to the work, practice and workplace vision. According to Manley, (2000) when there is a match between what we say we believe and what we do, it is one of the hallmarks of effective individuals, teams and organisations. Knowing what our values and beliefs are, is only the beginning of the process of positive change. A VCE tool is therefore useful when starting a journey of cultural change, or as a way for developing a common vision. Developing a common vision is a behaviour associated with transformational leadership (Manley ,1997).

Putting values and beliefs into practice involves overcoming barriers which exist in both us (whether as either competency, capability or capacity issues) and our workplaces (for example, experienced as culture, structures, processes, practices). Dismantling these barriers and recognising the gaps between what we say we believe, or what we say we do and what we do in practice. Becoming more self-aware and informed is characteristic to an emancipatory approach to practice development based on critical social science (Freire, 1985 ; Fay, 1987) and one which claims to achieve sustainable change (Manley and McCormack, 2003).

### 2.1.2. What does a values clarification exercise look like?

A values clarification exercise^[[1]](#footnote-2)^ (VCE) is a grand title for a simple exercise designed to access and clarify the values and beliefs we hold about something. The VCE uses a number of stem questions which can be adapted depending on the area you are focusing on with your stakeholders. Key to developing and realising a common shared vision is the involvement of as many stakeholders as possible in the process.

An example of this is: When developing an understanding of caring for older people within a unit or ward, the following stems may be useful:

- I believe the ultimate purpose of the caring for people on our ward/ unit is ........
- I believe this purpose can be achieved by ........
- I believe the factors that inhibit or enable this purpose to be achieved include: ........
- Other values/beliefs that are important are….

### 2.1.3. Who will be involved?

The VCE aims to create a shared vision and explicit ways of working among staff working on the ward. The VCE will be used at the start of the PAR/PD intervention (first three months) and form part of the creation of the purpose/vision, which will be constantly referred to over the THEO study period. VCE will also be used to critique the gaps between the values and those values that are experienced by the staff and the patients in the unit. The values experienced by staff and patients will be gathered using observation of care and emotional touchpoint interviews (see 2.6 and 2.7 below)

### 2.1.4. How will the VCE be used?

As it will be difficult to get all staff together at one time, the co-researchers will work with each other and small groups of staff (participants) at different times until as many people as possible have been involved in the process. This will involve asking everyone to answer the questions individually, these are then shared in the group and then the answers are collated. The participants will then gather the themes and look for similarities and differences. The group will then work together to use these themes to answer the following question:

- We believe the ultimate purpose of caring for older people within our unit is………

The participants may then choose to represent this as a statement or use creativity, by creating a poster for example. The THEO PD facilitator will then work with all the groups involved to pull this together into one common vision statement or representation for each ward/ unit.

### 2.1.5. What happens to the information arising from the values clarification exercise?

The information arising from using the VCE with as many stakeholder groups as possible is first collated before identifying common themes. Then through the process of collaboration these themes may inform:

- future strategic direction by converting themes about purpose into strategic vision statements/aims
- a position statement about shared values and beliefs
- the way work is organised so that it reflects the values held
- the structures and processes necessary to enable the strategy to become a reality.
- a curriculum or research framework
- the critique of practice at an individual/team level to establish the gaps between the values spoken about and the values experienced or observed.

### 2.1.6. VCE References

Fay, B., 1987. Critical social science: Liberation and its limits.

Freire, P., 1985. *The politics of education. Culture, power, and liberation*. Macmillan.

Manley K (2000) Organisational culture and consultant nurse outcomes: part 1 organisational culture. *Nursing in Critical Care*, Jul-Aug;5(4):179-84.

Manley K (1997) A Conceptual Framework for Advanced Practice: An action research project operationalising: An Advanced Practitioner/Consultant Nurse Role. *Journal of Clinical Nursing* 6(3) 179-190.

Manley K; McCormack B (2003) Practice development: purpose, methodology, facilitation and evaluation. *Nursing in Critical Care*, (8)1 :22-29.

Warfield C; Manley K (1990) Developing a new philosophy in the NDU. *Nursing Standard*, 4(41):27-30.

## 2.2. Pre and post leadership assessment for individual participants

This section explores the process of self-assessment of leadership styles, using the Guiding Lights for Leadership through an appreciative 360-degree assessment. We anticipate this section will take 10-20 minutes to read. However, you will be facilitated to use the approach by the THEO PD facilitator as part of the process of evidence capture for THEO in your role as co-researcher.

### 2.2.1. Introduction

Jackson et al. (2021) identify one of the key challenges facing all NHS organisations is to nurture cultures that ensure effective leadership for achieving the delivery of continuously improving high quality, person-centred, safe and compassionate care (West et al.,2015). Leadership is seen as one of the most influential factors in shaping organisational culture, so ensuring the necessary leadership behaviours, strategies and qualities are developed remains fundamental (Mannion et al., 2005). This message is reinforced by NHS England’s Reducing Length of Stay, where one of the key priorities is *enhanced clinical leadership*.

Jackson et al. (2021) work refined five guiding lights of leadership, as a metaphor for principles that enable and strengthen leadership across a range of contexts. These are:

- The Light Between Us - as interactions in our relationships
- Seeing People’s Inner Light
- Kindling the Spark of light and keeping it glowing
- Lighting up the known and the yet to be known
- Constellations of connected stars

This framework was developed and tested further, so that it can be used for several different purposes. The guiding lights of leadership can be used to frame the impact of leadership at three different levels – impact on self and others; impact on team(s)/workplace(s); impact on the system/ communities, regionally, nationally or internationally. This can be achieved by:

- Self-assessment for the purpose of guiding the practitioner in their professional and or career development.
- Demonstrating how the practitioner’s leadership role contributes to or supports impact with others.
- Contributing to academic or professional accreditation and or professional revalidation.
- Facilitating continuing inquiry into the practitioner’s own effectiveness of their leadership practice.
- Further development of leadership programmes that are based on sound theoretical principles.

### 2.2.2. How the leadership self-assessment tool will be used?

The tool will be used by the co-researchers in THEO to assess and develop themselves as transformational and collective leaders.

### 2.2.3. Who will be involved and when will it be used?

The leadership self-assessment tool will be used at the start of the PAR intervention and again at the end of the study period by all the co-researchers. The evidence gathered from using this self-assessment will be:

- used for personal and professional development purposes
- will be shared and analysed collectively to inform future activities
- will inform agreed steps for enhancing leadership potential to sustain improvements and cultures of effectiveness beyond the study period.

### 2.2.4. The leadership self-assessment tool

Jackson et al. (2021) provide the following information for achieving a self-assessment process as a process of appreciative feedback. See form 1 below.

| **Appreciative Feedback for Me. Name:** |
| --- |

I would be grateful if you could consider the following questions and make comments. (If there are questions you do not feel you can answer then leave blank). Do try to be as honest as you can to help me to learn and develop. If it is more helpful to discuss this with me rather than sending the form – please let me know. Try to think about specific examples in your feedback. Thank you in anticipation.

| **Area of exploration** | **Feedback** |
| --- | --- |
| **Guiding Light 1: The light between us as interactions in our relationships** | |
| What feedback would you like to give me about the way that I communicate with you? |  |
| What feedback would you like to give me about the way I communicate with and engage others? |  |
| Have you seen me being courageous at work? If so, what was this? |  |
| **Guiding Light 2: Seeing people’s inner light** | |
| What aspects about how I am at work do you think people value? |  |
| What would you say about how I am with emotions, my own and others? |  |
| How do I support people during stressful or emotional experiences? |  |
| How do you think I respond to difficult or sensitive situations with others? |  |
| In what ways do I give everyone a chance to participate or feel included? |  |
| What do I do to help create a safe environment for everyone to flourish? |  |
| What would you say about my ability to take on board other people’s perspectives? |  |
| **Guiding Light 3: Kindling the spark of light and keeping it glowing** | |
| What feedback can you give me about my ability to notice and build on people’s strengths? |  |
| What would you say about my ability to work with and collaborate with others? |  |
| What would you say about my ability to show support and appreciation to people? |  |
| Can you give me an example when you have noticed that I have shared learning/new insights with others? |  |
| **Guiding Light 4: Lighting up the known and the yet to be known** | |
| What feedback can you give me about my ability to remain calm and steady in complex and unpredictable situations? |  |
| When I am communicating with you and others, what would you say about my ability to hold off in making assumptions and ask questions? |  |
| What would you say about my ability to help people to come up with their own ideas? |  |
| What would you say about my ability to constructively challenge or stretch people? |  |
| What feedback can you give me about how I am and how I enable others to be flexible and creative with change and complexity? |  |
| **Guiding Light 5: Constellations of connected stars** | |
| What do I do that helps to build networks? |  |
| What feedback would describe how I connect internally and external with others to achieve collective action? |  |

| **General** | |
| --- | --- |
| If you could choose one word to describe me, what would it be? |  |
| If there was one thing that you feel I could do more of what would this be? |  |

Form 1 : Self-Assessment of Leadership via Appreciative Feedback (Jackson et al, 2021)

Please could you complete this form by XXX and email/send this this back to XXXX at ……

### Leadership Self-Assessment References

Jackson, C., McBride, T., Manley, K., Dewar, B., Young, B., Ryan, A. and Roberts, D., 2021. Strengthening nursing, midwifery and allied health professional leadership in the UK–a realist evaluation. *Leadership in Health Services*, *34*(4), pp.392-453.

Jackson, C., Manley, K. and Vibhuti, M., 2022. Change starts with me: an impact evaluation of a multiprofessional leadership programme to support primary care networks in the South East of England. *Leadership in Health Services*, 35(3), pp.309-337.

Mannion, R., Davies, H.T.O. and Marshall, M.N. (2005), “Cultural characteristics of ‘high’ and ‘low’

performing hospitals”, *Journal of Health Organization and Management*, Vol. 19 No. 6, pp. 431-439.

West, M., Armit, L., Eckert, R., West, T. and Lee, A. (2015),“Leadership and leadership development in healthcare: the evidence base”, available at <https://eprints.soton.ac.uk/439515/> accessed 13/03/24)

Permissions:

*360-degree feedback form by Dewar (2011) is licensed under the Creative Commons Attribution-Non-Commercial-Share Alike 4.0 International License. To view a copy of this license, visit* [*http://creativecommons.org/licenses/by-nc-sa/4.0/*](http://creativecommons.org/licenses/by-nc-sa/4.0/)*.*

## 2.3. Pre and Post Workplace Culture Assessment

We anticipate this section on undertaking a workplace culture assessment will take 10-20 minutes to read. However, you will be facilitated to use the approach by the THEO PD facilitator as part of the process of evidence capture for THEO in your role as co-researcher.

### 2.3.1. Introduction

A simple definition of workplace culture is ‘how things are done around here’ (Drennan 1992). Culture can be recognised as the behaviours, patterns and rules that become accepted as the norm. These are often “taken for granted” by the people working and being cared for within these workplaces. High-profile reports regarding Mid Staffordshire NHS Foundation Trust (Francis 2013), University Hospitals of Morecambe Bay NHS Foundation Trust (Kirkup 2015) and Liverpool Community Health NHS Trust (Kirkup 2018) have repeatedly brought to the fore the importance of culture in its widest sense and the multifactorial elements that contribute to organisational wide falling standards of accepted care. Mannion and Davies (2018 ,pg1) acknowledged that ‘*although culture is often identified as the primary culprit in healthcare scandals, with cultural reform required to remedy failings, such simplistic diagnoses and prescriptions lack depth and specificity’*. In other words, there is no guidance in how to work with workplace culture. One of the first places to start is to identify and understand the “taken for granted” behaviours, patterns and rules of a workplace culture. This can be done in a variety of ways depending on the context of each workplace culture and the people involved. A simple approach is to ask staff “what’s it like to work around here”. Collecting the narrative answers from a wide range of staff within a ward/ unit, collectively analyse and theming the answers will then form the basis for a collective action plan for improvement and help to identify the barriers and gaps between the “vision” and the “reality” of care provided on a ward/ unit.

### 2.3.2. How the workplace culture assessments will be used and who will be involved?

This exercise is aimed at the THEO co-researchers only and will involve undertaking activities to explore the culture of the ward and team at the start and end of the intervention and will be supported by the THEO PD Facilitator. This assessment aims to explore the workplace culture in the THEO implementation ward. There are a range of assessment activities for this purpose and collectively, the ward will be encouraged to choose a suitable assessment activity for this exercise.

### 2.3.3. Workplace Culture Assessment activities

#### 2.3.3.1. Visioning Exercise of the current workplace culture

Creating a psychologically safe space to explore culture requires time spent in achieving agreement to undertake the exercise as a negotiated process at the outset. Through creating a safe space for critical reflection, agreed ways of working, and ground rules clarifying expectations of each other’s behaviours as a process of mutual respect is achieved. It is important that you participate in a way that feels right for you - do your own thing. Using creative imagination is an approach to creating a vision of your workplace culture. This approach will help tap into both left and right side of the brain, as a mechanism to help address things that may be difficult to talk about or put into words, because it is deeply ingrained or experienced as a taken for granted activity, and not normally spoken about.

Spend a few minutes exploring whatever comes into your imagination, imagining the workplace culture with all your senses. Try to go with whatever comes into your imagination however bizarre at this point, and do not try to analyse it. Our imagination often holds just the right message.

#### 2.3.3.2. THEO PD Facilitator ‘script’/ guidance notes for a creative exploration of workplace culture

Start with inviting participants to engage in a grounding exercise which will help centred people in the ‘here and now’ and be truly present.

First make yourself comfortable – find a comfortable space in the room – this could be sitting or lying down; now I am going to invite you to close your eyes and listen to the sounds outside the room. We are going to leave the outside world for a time, letting go, for now, our thoughts about all our responsibilities at work and at home and our hopes, fears and expectations for this visioning session. Now bringing our attention to the sounds within the room ……... and now to the sounds within ourselves …… listening to the breath ……. and feeling the rise and fall of the chest …... letting go as we breathe out ….

Now imagine you are invisible, and you are going to your workplace today. Imagine how you are travelling there – by car, by bus, or walking …… Imagine you are entering the front of the building …. and then perhaps walking down a corridor, or upstairs , or in a lift …... passing through more door until you arrive at your workplace ….

When you get there, imagine moving through your workplace unnoticed.

- What do you see ….?
- What do you hear ….?
- What do you taste ….?
- What do you smell ….?
- What do you touch ….?
- What emotions do you experience …...?

And now it is time to leave, so imagine that you are turning around and moving towards the exit of your workplace, retracing your steps through doors …. down corridors …. steps or lifts …., until you reach the outside and you can feel the fresh air and hear the sound so the outside.

When you are ready, gently open your eyes … then, spend a few minutes alone to make a few notes, or drawings about what you experienced.

Let’s discuss what we have identified and how to then identify key elements or priorities that would improve the workplace culture.

#### 2.3.3.3. Bates’ Teams Culture tool

- Think about the culture of **YOUR TEAM**. Your team could be the staff you work with on a ward or in a community-based team.
- Read through the list (a. to l.) below and circle the number on each question that identifies the nearest to where you think **YOUR TEAM** is.

This questionnaire should take from **5 - 15 minutes** to complete. This tool is completed individually and then discussed collectively. A record can be made at any stage and used in a variety of ways. However, it is used, it important the same process is used at the beginning and repeated at the end of the THEO intervention.

**Table 2 Bates’ Team Culture Tool Questionnaire : Dewing & Pritchard, 2000)**

| 1. People in my team have dissimilar values, interests and beliefs | 1 | 2 | 3 | 4 | 5 | People in my team share values, interests and beliefs |
| --- | --- | --- | --- | --- | --- | --- |
| 1. People in my team break rank and go it alone | 1 | 2 | 3 | 4 | 5 | People in my team pull together |
| 1. Individuals in my team operate alone and there is conflict between them | 1 | 2 | 3 | 4 | 5 | There is community spirit and co-operation in my team |
| 1. My team is ruled by standards of the past | 1 | 2 | 3 | 4 | 5 | My team is ruled by visions of the future |
| 1. Meetings are an aspect of the culture in my team | 1 | 2 | 3 | 4 | 5 | Working in small teams is an aspect of the culture in my team |
| 1. In my team there are winners and losers, them and us | 1 | 2 | 3 | 4 | 5 | People confront and move beyond their differences in my team |
| 1. My team is anti-change | 1 | 2 | 3 | 4 | 5 | My team is change oriented |
| 1. There is weak co-ordination in my team | 1 | 2 | 3 | 4 | 5 | There is strong co-ordination in my team |
| 1. My team is inward looking and is focused on itself | 1 | 2 | 3 | 4 | 5 | My team is outward looking and does not focus on itself |
| 1. My team is dominated by routine and systems | 1 | 2 | 3 | 4 | 5 | My team is creative, and ideas dominated |
| 1. People do not reflect about their work in my team | 1 | 2 | 3 | 4 | 5 | People reflect about their work in my team |
| There is disagreement in my team | 1 | 2 | 3 | 4 | 5 | There is harmony in my team |

#### 2.3.3.4. The Fifteen-Step Challenge

The fifteen-step challenge was developed by the NHS Institute for Innovation and Improvement (2012) as part of the productive ward series. The basic tenant of the tool is that within the first 15 steps of entering a ward/unit an observer can experience the workplace culture. The tool kit includes preparing for the 15-step challenge, a recording tool, and how to create action plan.

### Workplace Culture Assessment References

Bates Team Culture Tool: Dewing J & Pritchard E (2000) Dewing, J., McCormack, B. and Titchen, A., (2014) Practice development workbook for nursing, health and social care teams. John Wiley & Sons. Pg 109

Drennan D (1992) Transforming Company Culture. McGraw-Hill, London.

Foundation of Nursing Studies(2015) Culture Change resources <https://www.fons.org/learning-zone/culture-change-resources/whatisculture> ( last accessed 14/3/24)

Francis R (2013) Report of the Mid Staffordshire NHS Foundation Trust Public Inquiry. Executive Summary. assets.publishing.service.gov. uk/government/uploads/system/uploads/ attachment_data/file/279124/0947.pdf

Kirkup B (2015) The Report of the Morecombe Bay Investigation. assets.publishing. service.gov.uk/government/uploads/ system/uploads/attachment_data/ file/408480/47487_MBI_Accessible_v0.1.pdf (Last accessed: 11 February 2022.)

Kirkup B (2018) Report of the Liverpool Community Health Independent Review. www.england.nhs.uk/wp-content/ uploads/2019/09/LiverpoolCommunityHealth_ IndependentReviewReport_V2.pdf

Mannion, R, Davies, H. Understanding organisational culture for healthcare quality improvement. BMJ 2018; 363:

NHS Institute for Innovation and Improvement (2012) The Fifteen Steps Challenge Quality from a patient’s perspective Part of the Productive Care resources. Online. Available from: [66269 PW Fifteen steps challenge new_Layout 1 (hic.org.au)](https://dev.hic.org.au/wp-content/uploads/2019/10/Health-Issues-Centre-15-steps-challenge-toolkit.pdf). Last accessed: 19/08/2024

Sanders K, Webster J, Cardiff S, Manley K (2021). Recognising & Developing Effective Workplace Cultures across Health & Social Care that are good places to work (Book Chapter) *In:* Manley, K., Wilson,V., Øye C. (Eds) *International Practice Development in Health and Social Care*. Wiley Press. Chichester.

Webster J, Sanders K, Cardiff S, Manley K. 'Guiding Lights for effective workplace cultures': enhancing the care environment for staff and patients in older people's care settings. Nursing Older People. 2022 May 31;34(3):34-41. doi: 10.7748/nop.2022.e1377. Epub 2022 May 4. PMID: 35506341.

## 2.4. Fourth Generation Evaluation – Claims, concerns and issues exercise (Guba and Lincoln, 1989)

We anticipate this section on undertaking the claims, concerns and issues exercise (Fourth Generation Evaluation) will take 5-10 minutes to read. However, you will be facilitated to use the approach by the THEO PD facilitator as part of the process of evidence capture for THEO in your role as co-researcher.

### 2.4.1. Introduction

Claims, concerns and issues (CCIs) (adapted from Kock, 1994) is a useful way in which you can gain the views and perspectives of all the people (stakeholders) who will be involved in and/or affected by the work that you are planning to undertake. It enables people to feel listened to be able to find the solutions and be part of how practice is improved.

- **A claim** is a positive statement or assertions that someone would make about a subject. These are any that a stakeholder may make that are favourable.
- **A concern** is a negative statement or assertion that someone would make about the same subject. The purpose is to highlight potential barriers: personal, systematic or organisational, real or perceived.
- **An issue** is a reasonable question about the subject; these are raised through a better understanding of the claims and concerns and are drawn from the latter by using ‘what’ and ‘how’ questions
- **Actions** are developed in response to the issues (questions) that are raised

CCIs comes from Fourth Generation Evaluation (Guba and Lincoln, 1989), an evaluation methodology that captures the opinions of stakeholders and uses these to plan on-going activity. So, in essence it is an approach to enable the development of shared action plans, but if these are used regularly, they become an evaluation approach, as actions that are identified are realised as claims over a period of time. An example of using CCI is described by Williams et al. (2018) in an action research workshop to explore standards for peri-operative nursing.

### 2.4.2. How and when the CCI will be used and who will be involved ?

The CCI’s will be facilitated regularly throughout the THEO intervention from month 1- month 11. This will involve the co-researchers primarily who will invite wider participation from the ward staff and organisational stakeholders who has a strong connection and influence on the ward where the THEO intervention is taking place. This activity will also be used as part of any shared learning events (see section 2.9) between the two participating wards in THEO. This will be facilitated in the following stages:

- Each participant is asked to write down all their claims and concerns about a topic
- The claims and concerns are then shared and collated
- The participants then collectively develop the issues/questions from the claims and concerns shared. These are then described using “ how, what, when , where” questions”
- The participants then collectively develop answers and solutions to the issues/questions. Then collective action plans are created from the answers. These shared action plans are used to progress change and improvements but also offer the opportunity to evaluate progress of those action plans in real time.

### 2.4.3. The CCI activity

| What claims or positive statements would you make about x? |
| --- |
|  |
| What concerns or negative statements would you make about x? |
|  |
| What issues / questions do you have about x? |
|  |
| What actions/ solutions do you have to the issues/ questions raised above? |
|  |

### CCI References

Guba, E.G. and Lincoln, Y.S. (1989) Fourth Generation Evaluation. Newbury Park, CA: Sage Publications.

Foundation of Nursing Studies (2015)*Culture change resources. Creating shared action plans* [Microsoft Word - CCIs.docx (fons.org)](https://www.fons.org/resources/documents/Creating-Caring-Cultures/CCIs.pdf)( last accessed 14/3/24)

Koch, T. (1994) Beyond measurement: fourth-generation evaluation in nursing. *Journal of Advanced Nursing* 20, 1148–115

Williams, C., Duff, J., Nicholson, P., Hamlin, L. and Gillespie, B.M., 2018. Using the ACORN Standards: An exploration of claims, concerns and issues. *Journal of Perioperative Nursing*, *31*(4), pp.37-41.

## 2.5. Staff and Patient Experience Interviews using Emotional Touchpoints

We anticipate this section on undertaking staff and patient experience interviews using emotional touch points will take 5-10 minutes to read. However, you will be facilitated to use the approach by the THEO PD facilitator as part of the process of evidence capture for THEO in your role as co-researcher.

### 2.5.1. Introduction

The emotional touchpoints method focuses on asking people to describe an experience using a selection of words that describe an emotion. People are asked to think about key points in their experience and to select from a range of emotional words that best describes how they felt at the time. The method helps the interviewer and interviewee to directly focus on the emotion related to the different points (touchpoints) in the experience. This approach was originally designed to enable patients and families to share their experiences but has been adapted to enable a variety of experiences to be shared. Evidence suggests that these subjective experiences are an effective way of exploring and understanding practice, enabling celebration and stimulating innovation and development (Bate and Robert, 2007; Dewar and Noble, 2013).

### 2.5.2. Emotional Touchpoints websites (free access)

We have provided links to the Healthcare Improvement Scotland and Alzheimer’s Society websites which provide detailed information about the emotional touchpoint process, including how to undertake an interview with patients and staff and a video of this process in action. Please see section 2.5.4 for these websites.

### 2.5.3. How will the approach be used?

The emotional touchpoint approach will be used by the co-researchers to understand their own experiences of the ward within the THEO intervention. Then wider participation will also be sought by inviting patients and staff within the ward/unit to share their experience of care and to gain evidence about what is happening in practice on the ward/unit and help the co-researchers identify the gaps between “what we say” and “what we do”. The number of people recruited will be negotiated by the co-researchers based on the context in which they are working. It is anticipated that between 3- 6 staff participant interviews will be undertaken and 1-3 patient and personal consultee interviews will be undertaken. These will be undertaken in month 4-6 of the THEO intervention.

Relevant participant information sheets (staff, patients and where applicable, their personal consultees) will be shared with potential participants to inform them what the interview is about, their rights and how we will use any information they provide during the interview. Once they have read, understood and clarified any queries they might have, participants will be required to complete a consent form after which the interview will be scheduled for a mutually agreed time and location. Interviews will be conducted face-to-face in a private location on the hospital ward, although we recognise that some patients may not be well enough to mobilise to another space for interview purposes, in which case, interviews will be conducted by their bedsides. Participants on muti-occupancy bays/rooms will have curtains drawn to ensure their privacy while those in single rooms will have interviews conducted in their rooms. For patients lacking mental capacity, interviews will take place with their personal consultees in a secure location within the hospital, or by the patients’ bedsides, depending on their preferences and unique circumstances. Interviews will be recorded on an audio-device, and may last up to an hour, depending on the level and depth of the discussion.

### Emotional Touchpoints References

Alzheimer’s Society (n.d.) Emotional Touchpoints. Available from: <https://www.alzheimers.org.uk/dementia-professionals/dementia-experience-toolkit/research-methods/emotional-touchpoints>. Last accessed : 15^th^ August 2024.

Bate, P. and Robert, G. (2007) *Bringing User Experience to Healthcare Improvement*. Oxford: Radcliffe Publishing

Brodie J (2021) Using ‘emotional touchpoints’ to support staff during Covid-19. Nursing Times [online]; 117: 12, 23-25. [Using ‘emotional touchpoints’ to support staff during Covid-19 | Nursing Times](https://www.nursingtimes.net/clinical-archive/wellbeing-for-nurses/using-emotional-touchpoints-to-support-staff-during-covid-19-08-11-2021/)

Dewar, B., Mackay, R., Smith, S., Pullin, S. and Tocher, R. (2009) Use of emotional touchpoints as a method of tapping into the experience of receiving compassionate care in a hospital setting. *Journal of Research in Nursing*. Vol. 15. No. 1. pp 29-41

Dewar, B. and Noble, M. (2013) Caring about caring: developing a model to implement compassionate relationship centred care in an older people care setting*. International Journal of Nursing Studies*. Vol. 5. No. 9. pp 1247-1258

Foundation of Nursing Studies (2015) *Culture change resources: Using Emotional Touchpoints to Understand Experiences of Care.* [Microsoft Word - EmotionalTouchpoints .docx (fons.org)](https://www.fons.org/resources/documents/Creating-Caring-Cultures/Emotional-Touchpoints.pdf) ( Last accessed 14/3/24)

Healthcare Improvement Scotland (2020) Emotional touchpoints. Available from: <https://www.hisengage.scot/equipping-professionals/participation-toolkit/emotional-touchpoints/>. Last accessed: 15^th^ August 2024

## 2.6. Observation of Care – Workplace observations

We anticipate this section on undertaking an observation of care- workplace observation will take 5- 10 minutes to read. However, you will be facilitated to use the approach by the THEO PD facilitator as part of the process of evidence capture for THEO in your role as co-researcher.

### 2.6.1. Introduction

Observing care as it is delivered, and learning from these observations can be a basis for quality improvement (Royal College of Nursing, 2004). The Foundation of Nursing Studies (FoNS, 2015) describe that an Observation of Care takes place over a short period of time (usually around 15-30 minutes) to observe the activities in an area where care is delivered and then feeding the observations back to the staff involved to identify learning and action points. It allows the observer to:

- See the reality of care (Underwood, 2014)
- Understand the environmental and emotional context in which care takes place
- Have an opportunity to look differently at the way care is organised – to celebrate what is done well and to identify what could be improved (FoNS, 2015)

### 2.6.2. How and when will the Observation of Care approach be used?

The observation of care approach will be used by the co-researchers in collaboration with the existing team members who will be recruited as participants, to observe activities and interventions of care on the ward , to gain evidence about what is happening in practice on the ward and help the co-researchers identify the gaps between “what we say” and “what we do”. The number of observations will be negotiated by the co-researchers based on the context in which they are working. It is anticipated that between 1 and 3 observations will be conducted. One or two people will undertake the observations and will be paired with someone who compliments their skills and experience. These will be undertaken between month 4 and 6.

The scheduling of observations will depend on the availability of the co-researcher team and clinical pressures on the ward. Therefore, instead of targeting specific activities, the observing co-researchers will focus on gaining a comprehensive understanding of ways of working and how tasks are performed on the ward. You will observe the practice of members of staff that have provided their consent to be observed, whilst they provide care for patients. Only verbal consent will be required for this activity. On the day of the observations, staff on shift will be requested to verbally inform patients and where applicable, their consultees that observations are being conducted on the ward. For patients lacking mental capacity, their consultees should be informed; preferably personal consultees but in instances where they are unavailable to provide immediate advice, their professional consultees should be informed. On the day of the observations, you will have an opportunity to talk through observing practice with staff members who have provided their consent to have their practice observed, so that they understand the process and the principles of giving effective feedback.

Patients will not actively participate in this activity, and they do not need to change their usual routine or do anything differently to if the observation was not being conducted. You will need to have obtained verbal consent to enter patients’ rooms or be by their bedsides. You also have a legal and ethical obligation to respect patients’ autonomy and privacy and leave their rooms or bedsides if asked to do so. For patients with lacking capacity, you are required to watch out for facial and verbal cues to check for distress or disagreement to being in the room or by their bedside and leave immediately if this happens. Patients and where appropriate, their personal consultees are free to verbally opt out at any time before or during the observations although it will not be possible to withdraw already collected observational data due to its anonymous nature once data analysis has commenced. Where a patient or their consultee opts out, this should be documented in their medical record. No personal data should be collected, and participants should not be identifiable from any observational data including any notes taken**.** On completion of the observations, you will have the opportunity to discuss with other co-researchers on what you have observed and identify what and how you are going to feedback to the team. The Foundation of Nursing Science (2015) offers a good preparation guide for conducting observations of care. Please see reference list in section 2.6.8.

### 2.6.3. Example of the Observation of Care tool for recording observation

Table 3: Adapted from Enhancing Care for Older People - A Guide to Practice Development Processes to Support and Enhance Care in Residential Settings for Older People. ISBN: 978-1-906218-35-5.

| **Name of observer** |  | **Unit** |  |
| --- | --- | --- | --- |
| **Focus of observation** |  | **Date** |  |
| **Time** | **Observation notes** | | **Observer comments/ questions** |
|  |  | |  |

### 2.6.4. Observation of practice feedback template

Name of Observer: ...........................................................................................

Unit: ...............................................................................................................

Focus of Observation: .........................................................................................

Date: ...............................................................................................................

Staff present at feedback session ..............................................................................

| **Observation data** | **Observer comments** | **Observer feedback** | |
| --- | --- | --- | --- |
|  |  | **Key areas being actioned (including immediate actions already taken)** | **Key areas for celebrating/ sharing** |
|  |  |  |  |

### 2.6.5. Principles of Giving and Receiving Feedback

We anticipate this section on the principles of giving and receiving feedback will take 5-10 minutes to read. However, you will be facilitated to use the approach by the THEO PD facilitator as part of the process of evidence capture for THEO in your role as co-researcher.

#### 2.6.5.1. Principles for giving effective feedback

Use the table below to consider what, how and why feedback is being provided. Information about this process can be found in the Practice Development Workbook **(**Dewing, J., McCormack, B. and Titchen, A., (2014) Pg 100)

| **CRITICISM is commonly experienced as:**   - Lack of interpersonal skills in the criticiser - Perceived as negative by receiver - Effects on the receiver/Negative outcomes e.g. destructive or unproductive - Criticises behaviour - Judgmental i.e. making judgements about the value of something - Focuses on the person - Disabling to the recipient - Criticiser is perceived as being harsh - Criticiser ‘jumps in with both feet’ i.e. hasn’t verified or checked out anything before criticising - Unstructured - Problem focused | **FEEDBACK is commonly experienced as:**   - Enabling - Positive e.g. constructive and planned - Specific Skills for giving feedback - Structured - Supportive - Positive effects on receiver - Considered response - Non- Judgmental - Action from\positive outcomes - Nature of feedback maybe negative as well as positive |
| --- | --- |
| **PRINCIPLES FOR GIVING AND RECEIVING FEEDBACK**   1. Get the facts right before giving feedback 2. Plan in advance for how you are going to give feedback 3. Prepare feedback carefully and practice 4. Identify appropriate methods for giving feedback 5. Encouraging staff to feel part of the process in advance so that it doesn’t feel a ‘them and us’ situation 6. To work with the clinical leader so she/he can help with the giving of feedback to ward teams 7. Encourage recipients of feedback an opportunity to share their own self-assessment first before giving feedback from patient stories e.g. asking, ‘What do you think patients are specifically saying about how they experience our service?’ 8. Give support before, during and after giving feedback 9. Give feedback on behaviour/issues not the person and their ‘personhood’ (i.e. soft on the person, hard on the behaviour/issues) 10. Enable increased self-awareness of behaviour and its consequences 11. Provide non-judgmental feedback, which is truthful, direct and constructive 12. Focus on how the receiver of feedback can be in a position to move forward 13. Provide follow-up 14. Enable recipients to give us feedback on how we gave the feedback and how it could have been more effective. 15. Provide an opportunity to enable staff to action plan based on feedback and provide opportunities to identify how to share good practices | |

### 2.6.6. Observation of Care: Frequently Asked Questions

**Q1: What will you do if you observe practice that causes you concern?**

- All registered practitioners have a duty of care to the patient. If practice is observed that causes immediate risk to the patient, then action will need to be taken to ensure that the risk is reduced even if this means discontinuing the observation activity.
- If the risk is not immediate, then depending on the observer’s judgement, acknowledgement of this may be able to wait until the end of the observation period.

**Q2: Do we need to obtain consent from patients?**

- Yes – although we are not directly observing patients but the care environment in which patients are positioned. However, it will be important to be sensitive to different patient’s needs (e.g. situations where patients are at the end of life, being resuscitated or where there are distressed relatives) and environments.
- It is important to inform and seek consent from patients, and where applicable, their consultees about what you are doing and if this causes concern to any patients. It will be important to act on these concerns.

**Q3: What do we do if there is an unconscious patient within the area being observed?**

- Unconscious patients are more likely to be in a critical care setting where there are a smaller number of beds and where observation area may only include 2-3 bed areas. The same principles apply as above.
- Observers would never go behind curtains and would position themselves so as to observe the 2-3 bed areas.
- Personal/professional consultees would need to be informed what you were doing if the patient couldn't be informed themselves.
- If distressed relatives were present or the patient became acutely unstable then it may be more sensitive to renegotiate the observation area.

***Remember....***

- Each area’s individual, patient and staff member is unique
- Using a co-observer provides another viewpoint
- Preparation is vital
- The whole process is open and transparent
- Building in time to debrief, feedback and reflect at all stages is vital
- Ensure you seek personal support for debriefing following a difficult observation of care and feedback.
- The importance of meeting with the Ward Manager and team to follow upon all observation

### Observation of Care References

Dewar, B. and Nolan, M., 2013. Caring about caring: Developing a model to implement compassionate relationship centred care in an older people care setting. *International journal of nursing studies*, *50*(9), pp.1247-1258.

Denner, L.,Thompson, L,.,Chambers, B., Jackson, D., Cooke, H., Magill, L., Bubb, J., Miller, J., Harries, M., Atkins, S., (2019) Observing everyday interactions to uncover compassion in care. *Nursing Times*, Vol. 115, Issue 1

Dewing, J., McCormack, B. and Titchen, A., (2014) *Practice development workbook for nursing, health and social care teams* pp 102-106

Foundation of Nursing Studies (FoNS) (2015) : Culture change resources: Observation of Care [Microsoft Word - ObservationOfPracticeResources.doc (fons.org)](https://www.fons.org/resources/documents/Creating-Caring-Cultures/ObservationOfPracticeResources.pdf) ( Last accessed 14/3/24)

Royal College of Nursing (2004) The RCN Clinical Leadership Toolkit. London: RCN.

Underwood F (2014) Practice question: how can observations of care help me improve my practice? *Nursing Older People*; 26:2,13.

## 2.7. Final Participatory Evaluation

### 2.7.1. Introduction

Hardy et al., (2021, pg. 113) state that “PD uses inclusive evaluation to integrate evidence from process and outcomes of transformation” then go onto to describe that the stories of all involved should be heard and included in the assessment summary and therefore is universal and electric in its evaluation approach. Odell (2018) describes a participatory evaluation that involves key stakeholders in answering several reflective questions individually, then working together to collectively share, analyse and theme and represent these themes creatively back to the group. You can draw evidence for the evaluation from your reflective journals and in addition you can look at appendix 2, which is preliminary PD table of evidence and consider what you have experienced as part of THEO intervention.

### 2.7.2. How and when will the evaluation be used?

The participatory evaluation is just one of the many sources of evidence that will be co-created and analysed and represented by the co-researchers towards the end of the intervention period together.

### 2.7.3. A series of reflective questions for individual use

1. What has been your key learning from participating in the THEO/ PD intervention?
2. What new skills have you learnt?
3. How has your confidence changed and what has this impacted on?
4. What changes/improvements have been introduced ?
5. What has changed in your leadership style ?
6. What aspects of participating did you find most useful and why?
7. What aspects could have been done differently and why?
8. What recommendations do you have for the future of THEO?

### 2.7.4. Keeping a reflective journal

Throughout the THEO project, we suggest that the co-researcher team keep a reflective journal of the project, to capture your thoughts, emotions, highs and lows of active engagement with a project of this nature. Using your journal to capture your reflections will be useful when meeting with the THEO PD facilitator, as a reminder of issues being raised in daily clinical practice, navigating relationships with colleagues, those snippets of enjoyment received and captured when things go well.  It will also be a useful reminder of the journey or process of the THEO intervention and will provide useful evidence for the participatory evaluation. The reflective journals will remain the property of the co-researchers and remain private unless they choose to share evidence from them at various points of the THEO intervention, which forms part of the PAR.

Reflective journaling has multiple benefits. Multiple online search sites offer advice, tips and guidance on writing and keeping reflective journal entries. You can choose a reflective tool from the reference below and work through the aspects of what happened, when and where did this occur, what did you/others do, how did you feel, what have you learned, what might you do differently?

*Rolfe, G., Freshwater, D., Jasper, M. (2001) Critical reflection in nursing and the helping professions: a user’s guide. Basingstoke: Palgrave Macmillan*

*Kessler, Penny D. MN, RN; Lund, Carole H. PhD, APRN, BC. (2004) Reflective Journaling: Developing an Online Journal for Distance Education. Nurse Educator 29(1):p 20-24.*

### Final Participatory Evaluation References

Hardy, S., Clarke, S., Frei, I. A., Morley, C., Odell, J., White, C. & Wilson, V. (2021) A Global Manifesto for Practice Development. In Anonymous *International practice development in health and social care.* 99-117.

Odell, J., 2018. Reflections on developing a participatory evaluation as part of the Patients First programme. *International Practice Development Journal*, 8(2).

## 2.8. Shared Learning Events

### 2.8.1. Introduction

As the Theo intervention (PAR) progresses, various shared learning events will be used to:

- Bring together the two clinical team co-researchers to share activities and outcomes to date, across sites.
- Share findings with a wider stakeholder group from the ICS and gain different perspectives on the knowledge generated and actions identified
- These events will involve using the fourth-generation evaluation activity ( CCI, see section 2.5) to generate further evidence and to contribute to that generated for the PAR.

### 2.8.2. When will this happen?

The events will be co-created and negotiated as the THEO intervention (PAR) progresses. The timing and number of events will be influenced by the context of each unit and the ability for the co-researchers to be released from clinical practice. However, it is anticipated that there will be one midway through PAR and one at the end that will also be a celebration event.

### 2.8.3. Who and what will be involved?

As these events will be co-created these will be designed and attended by the co-researchers and a wider stakeholder group by negotiation.

# Section 3: Closing the intervention phase of THEO

## Lessons Learned

The final stages of the intervention phase will involve working together to bring the participatory relationships to a close. The intention will be to consider where and how further integration and dissemination can be achieved across the system. A celebration of achievements will be co-created by the co-researchers and be used for spreading innovation, and ensuring the wider system partners are also able to share best practice and dissemination of findings will be encouraged through conventional routes such as conference papers and professional/academic publications, as co-authors with participants.

# Appendix 1: Table showing the THEO implementation activities and when they will occur during implementation of the THEO intervention

|  |  |  | Month | | | | | | | | | | | |
| --- | --- | --- | --- | --- | --- | --- | --- | --- | --- | --- | --- | --- | --- | --- |
| **Activity** | **Who** | **How long each time** | **1** | **2** | **3** | **4** | **5** | **6** | **7** | **8** | **9** | **10** | **11** | **12** |
| Values clarification exercise | Co-researchers and ward staff | Varied - 1 – 3 hours |  | | |  |  |  |  |  |  |  |  |  |
| Leadership assessment | Co-researchers only | Varied - 1 – 3 hours |  | | |  |  |  |  |  |  |  |  |  |
| Workplace culture assessment | Co-researchers only | Varied - 1 – 3 hours |  | | |  |  |  |  |  |  |  |  |  |
| Emotional touchpoint interviews | Co-researchers, ward staff and patients and families | Up to 1 hour |  |  |  |  | | |  |  |  |  |  |  |
| Observations of care | Co-researchers, ward staff and patients and families | Up to 20 minutes |  |  |  |  | | |  |  |  |  |  |  |
| Fourth generation evaluation (CCI) | Co-researchers, ward and organisation staff | Varied – 1 to 6 hours | X | X | X | X | X | X | X | X | X | X |  |  |
| Final participatory evaluation | Co-researchers only | Varied – 1 to 6 hours |  |  |  |  |  |  |  |  |  | X |  |  |

# Appendix 2 : Preliminary theory of change/logic model and associated PD evidence source

| **Inputs** | **Activities** | **Outputs** | **Outcomes/Consequences** |
| --- | --- | --- | --- |
| Practice Development (PD-as a complex intervention (McCormack et al., 2013) | Skilled facilitation of action cycles (Crisp & Wilson, 2011)  Baseline assessment in collaboration with stakeholders to build PD interventions upon.  Collaboration, inclusion, and participation to codesign improvement activities (Hardy et al., 2021)  Working with context, culture, and unpredictability.  Forging strong, collaborative, authentic relationships as an ‘outsider’ working and navigating practice, organisation, and system complexity. | Shared (clear) purpose framework (Manley et al., 2014)  Care that is wrapped around the person (Manley et al., 2023)  Transparent PD processes to support learning, development, and transformation.  Thinking and working that reflects critical reflection and reflexivity ‘in’ and ‘on’ practice.  Seeks out opportunities to reflect and live the value of person-centred relationships.  Links between ‘optimisation’ and PD Interventions are visible, seen and articulated. | Person centred practice (McCormack & McCance, 2017)  Workplace culture of effectiveness/sustained improvement and ability to adapt to change (Webster et al., 2022a)  Changes linked to the base line assessment are seen and sustained.  A sustained workplace culture of ongoing learning, development, and transformation. |
| Multidisciplinary teamwork | Effective teamwork to achieve safe effective patient care (Manley et al., 2019) | Respectful communication and appreciation of expertise from whole team  Role clarity (King et al., 2021)  Opportunities for shared learning are sought and valued.  Articulation of person-centred outcomes linked to interdisciplinary team working. | Patients made central to decision making and recognised as an expert by lived experience.  Skills and attributes used collectively linked to outcomes and therapeutic optimisation.  Enhanced work experience /wellbeing (Sanders et al., 2021), professional growth and development. |
| Clinical Leadership (Manley & Titchen, 2017) | Effective and collaborative leadership, including willingness to delegate (Akhtar et al., 2016) and manage risk.  Opportunities for shared learning are sought out, valued, and established. | A creative, inclusive approach to solution focused patient care (Middleton, 2013) that is focussed on the ‘person’.  Maximising accountability to deliver care fit for purpose (Jackson et al., 2022) that is professionally led through shared governance and leadership. | Safe and effective clinical decision making that is embedded within shared governance. |
| Evidence based practice | Critical evaluation of evidence embedded in activities and decision making (Hardy et al., 2011) linking optimisation to PD interventions. | Evidence from both ‘in’ and ‘on’ practice is used to inform practice change, development, and ongoing learning.  Enhanced skills to address issues in ‘real time’ | Wider organisational/system sharing, learning and sustained innovation uptake. |

1. Warfield C; Manley K (1990) Developing a new philosophy in the NDU. Nursing Standard, 4(41):27-30. [↑](#footnote-ref-2)
