# Supplementary material for: Quasi-experimental study on the effectiveness and impact of implementing nurse-led ‘therapeutic optimisation’ (THEO) intervention in two older persons wards: a mixed methods study protocol
Source: BMJ Open. 2025 Aug 26;15(8):e102529. doi: 10.1136/bmjopen-2025-102529 (PMC12382520; doi:10.1136/bmjopen-2025-102529)
Supplement: Supplementary file 2 [file bmjopen-2025-102529.reviewer_comments.pdf]

PEER REVIEW HISTORY

BMJ Open publishes all reviews undertaken for accepted manuscripts. Reviewers are asked to complete a checklist review form and are provided with free text boxes to elaborate on their assessment. These free text comments are reproduced below.

ARTICLE DETAILS

Title (Provisional)

A quasi-experimental study on the effectiveness and impact of implementing nurse-led 'Therapeutic Optimisation' (THEO) intervention in two older persons wards: A mixed methods study protocol

Authors

Ataiyero, Yetunde; Dube, Alisen; Odell, Joanne; Carter, Vanda; Smith, Hazel A; Hardy, Sally; Leary, Alison; Jones, Sarahjane

---

VERSION 1 - REVIEW

**Reviewer**	**1**
**Name**	**Krause, Rene**
**Affiliation**	**University of Cape Town, FaCE**
**Date**	**13-May-2025**
**COI**	**None**

I am concerned that trustworthiness and reflexivity has not been discussed in the manuscript. Bias is a major concern in this research and this has to be addressed.

Review of: A quasi-experimental study on the effectiveness and impact of implementing nurse-led 'Therapeutic Optimisation' (THEO) intervention in two older persons wards: A mixed methods study protocol

The aims and objectives of the study are clearly articulated, and the methodological approach is generally appropriate and well-aligned with the research objectives. However, the protocol would benefit from a more detailed description of the study site. Such elaboration would enhance the contextual depth necessary for a Realist Review and aid in interpreting context-mechanism-outcome configurations.

A notable concern lies in the insufficient attention given to trustworthiness and reflexivity within the proposed research design. Given the embedded nature of the study, it is essential that strategies to mitigate potential bias are clearly outlined. Explicit steps to ensure the credibility and dependability of the research process—particularly in relation to the qualitative components—should be included.

Additionally, the protocol does not adequately address how trustworthiness will be ensured within the quantitative strand of the study. Clarification of procedures to maintain rigour, such as data validation, triangulation, and reliability checks, would substantially strengthen the proposal.

VERSION 1 - AUTHOR RESPONSE

**Comments**	**Our Responses**
**Reviewer 1: Dr. Rene Krause, University of Cape Town**	
The aims and objectives of the study are clearly articulated, and the methodological approach is generally appropriate and well-aligned with the research objectives. However, the protocol would benefit from a more detailed description of the study site. Such elaboration would enhance the contextual depth necessary for a Realist Review and aid in interpreting context mechanism-outcome configurations.	We have expanded the description of the study setting to provide richer contextual detail, which we agree is essential for supporting the interpretation of context–mechanism–outcome (CMO) configurations in realist research. Specifically, we now describe the demographic and geographic features of Norfolk and Waveney, highlighting relevant indicators of deprivation and health inequality. We have also clarified the nature and location of the two NHS Trusts involved in the study – one within a community NHS Trust in a central urban area and the other within an acute NHS Trust in a coastal region, to enhance transparency and contextual understanding.<br><br>**Revision added to page 6:**<br>*Norfolk and Waveney are considered high areas of deprivation and need (Norfolk City Council, 2019). As a result, research has highlighted the need to have greater investigations on health inequalities that are present in coastal and rural areas (Public Health England, 2019). The clinical care setting will focus on care for older persons, which reflects the demographic population of Norfolk and Waveney. One care site is situated in a community NHS Trust, in a central, urban area of Norfolk, and the second*

Comments	Our Responses
	site is situated in an acute NHS Trust in the East of Norfolk, identified as a coastal region.
A notable concern lies in the insufficient attention given to trustworthiness and reflexivity within the proposed research design. Given the embedded nature of the study, it is essential that strategies to mitigate potential bias are clearly outlined. Explicit steps to ensure the credibility and dependability of the research process—particularly in relation to the qualitative components—should be included.	We have now expanded the protocol to more explicitly address these concepts, especially in relation to the qualitative components of the Participatory Action Research (PAR), interview-based, and co-produced process evaluation activities.<br><br>To enhance credibility, we have outlined procedures including independent double-coding of qualitative data, triangulation across multiple data sources (e.g., interviews, observations, engagement logs), and iterative member checking during participatory workshops. These steps will ensure the accuracy of the data and promote a deeper understanding of the research context.<br><br>To address dependability, we have described the use of detailed audit trails, use of a PAR Handbook to guide consistent facilitation, and reflexive journaling by co-researchers to document evolving interpretations and insights. These strategies will ensure that the research process remains transparent and methodologically consistent.<br><br>We have also incorporated explicit reflexivity strategies, including facilitated reflexive spaces for co-researchers and the research team to critically examine their own positionality, assumptions, and potential influences on the research process. This reflective practice will enable a deeper understanding of how our perspectives may shape the research findings.

Comments	Our Responses
	Our approach in the qualitative work packages is inductive rather than deductive, as seen in the quantitative work packages. We prioritise knowledge co-creation with participants and stakeholders, allowing the research process to evolve dynamically in response to their input.<br><br>These additions are now described in the revised protocol under the "enhancing rigour in the qualitative components" section [page 25 – 26] and will support the methodological rigour of the study.<br><br>**Newly added text:**<br>"*To enhance the trustworthiness of the qualitative components, we will employ multiple strategies consistent with Lincoln and Guba's evaluative criteria (1985). Credibility will be supported through triangulation of data from diverse sources (e.g., staff, patients, observations), peer debriefing within the research team, and iterative member checking conducted during the co-production workshops. Within the workshops, co-researchers will undertake the theming and analysis of all gathered evidence, ensuring that the process is cross-checked by all team members. The THEO PD facilitator will be present to challenge any assumptions made by the team, ensuring a critical examination of the data. Co-researchers, who come from various roles within the ward team, will bring a range of perspectives, further enhancing the validity of the findings. In addition, the THEO intervention registered nurses, although embedded within the NHS Trusts, are new to the ward and bring a fresh set of eyes, offering an*

Comments	Our Responses
	element of independence that is valuable both from a trustworthiness standpoint and in terms of developing relationships that are essential for the success of the intervention.<br><br>*Dependability will be ensured by maintaining a comprehensive audit trail that documents methodological decisions, alongside detailed records of facilitation processes utilising the THEO PAR Handbook. These strategies will ensure that the research process remains transparent and methodologically consistent.*<br><br>*Reflexivity will be systematically embedded through scheduled reflexive discussions involving both the research team and co-researchers, aimed at critically examining the influence of our positionalities, assumptions, and potential biases. Co-researchers will be encouraged to maintain reflective journals throughout the intervention period. Where appropriate and with informed consent, selected excerpts from these journals will be incorporated to enrich data interpretation and facilitate critical engagement with the evolving context of implementation.*
Additionally, the protocol does not adequately address how trustworthiness will be ensured within the quantitative strand of the study. Clarification of procedures to maintain rigour, such as data validation, triangulation, and reliability checks, would substantially strengthen the proposal.	We agree that clearly articulating the procedures for maintaining rigour in the quantitative strand is essential to enhancing the robustness of the study. We have now revised the protocol to include details on:<br>• Data validation procedures, including pre-analysis data cleaning, completeness checks, and the use of data dictionaries;

Comments	Our Responses
	• Triangulation, by integrating findings from quantitative outcomes (e.g., nurse-sensitive indicators) with qualitative insights from interviews and participatory activities during the mixed methods process evaluation;<br>• Reliability checks, such as consistency testing of variables across timepoints and sites, as well as the use of validated instruments (e.g., NoMAD questionnaire) for implementation evaluation.<br>**Revisions are included under the "Enhancing rigour in the quantitative components" section on pages 26-27 of the manuscript.**
